# DNA methylation controls stemness of astrocytes in health and ischaemia

**DOI:** 10.1038/s41586-024-07898-9

**Published:** 2024-09-04

**Authors:** Lukas P. M. Kremer, Santiago Cerrizuela, Hadil El-Sammak, Mohammad Eid Al Shukairi, Tobias Ellinger, Jannes Straub, Aylin Korkmaz, Katrin Volk, Jan Brunken, Susanne Kleber, Simon Anders, Ana Martin-Villalba

**Affiliations:** 1 Division of Molecular Neurobiology, German Cancer Research Center (DKFZ), Heidelberg, Germany; 2 BioQuant Centre, University of Heidelberg, Heidelberg, Germany

**Keywords:** Neural stem cells, DNA methylation, Neural stem cells, Epigenetics and plasticity, Bioinformatics

## Abstract

Astrocytes are the most abundant cell type in the mammalian brain and provide structural and metabolic support to neurons, regulate synapses and become reactive after injury and disease. However, a small subset of astrocytes settles in specialized areas of the adult brain where these astrocytes instead actively generate differentiated neuronal and glial progeny and are therefore referred to as neural stem cells^1–3^. Common parenchymal astrocytes and quiescent neural stem cells share similar transcriptomes despite their very distinct functions^4–6^. Thus, how stem cell activity is molecularly encoded remains unknown. Here we examine the transcriptome, chromatin accessibility and methylome of neural stem cells and their progeny, and of astrocytes from the striatum and cortex in the healthy and ischaemic adult mouse brain. We identify distinct methylation profiles associated with either astrocyte or stem cell function. Stem cell function is mediated by methylation of astrocyte genes and demethylation of stem cell genes that are expressed later. Ischaemic injury to the brain induces gain of stemness in striatal astrocytes^7^. We show that this response involves reprogramming the astrocyte methylome to a stem cell methylome and is absent if the de novo methyltransferase DNMT3A is missing. Overall, we unveil DNA methylation as a promising target for regenerative medicine.

## Abstract (web summary)

Single-cell analysis of the transcriptome, chromatin accessibility and methylome of adult neural stem cells and astrocytes demonstrates that stemness is driven by methylation profiles distinct from those of astrocytes.

## Main

**Fig. 1:**
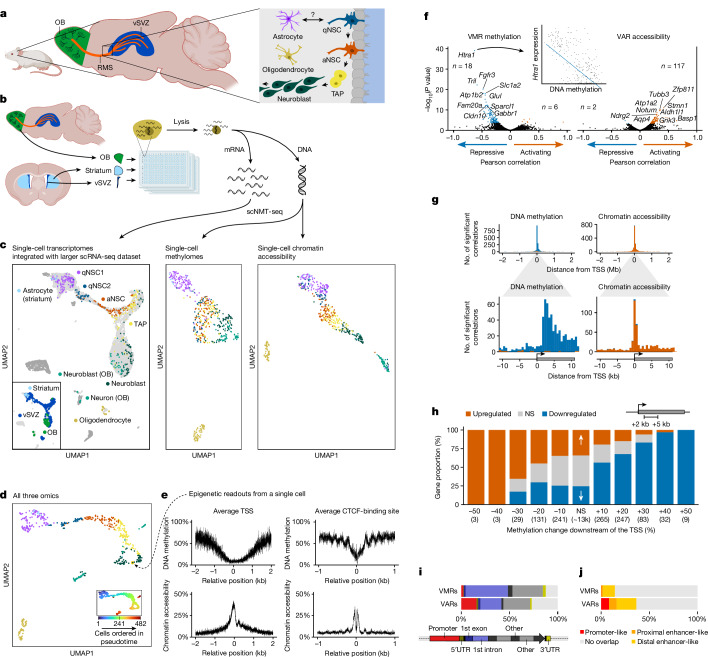
Single-cell triple-omics of the adult NSC lineage. **a**, Schematic depiction of the adult NSC lineage of the vSVZ. NSCs represent a specialized subset of astrocytes. OB, olfactory bulb. **b**, Schematic of the workflow to obtain scNMT-seq data from three brain regions. **c**, UMAP visualization of each molecular layer, colour coded as in **a**. Single-cell transcriptomes (540 cells from 5 replicates) were integrated with the larger dataset from ref. ^18^, which comprises cells from the vSVZ (light grey) and striatum, RMS and olfactory bulb (dark grey). Bottom left, tissue of origin for each cell. **d**, UMAP and pseudotime ranks (excluding oligodendrocytes) based on data from all three molecular layers. **e**, Average methylation and chromatin accessibility levels around TSSs and CTCF-binding sites of one individual neuroblast. **f**, Correlation of VMR methylation and VAR accessibility with expression of the nearest gene. Negative correlations (blue) indicate a repressive effect of methylation or accessibility; positive correlations (orange) indicate activation. *n* is the number of nominally significant correlations (Pearson’s two-sided correlation test) after Benjamini–Hochberg adjustment. The *y* axis shows unadjusted *P* values. **g**, Top, distance histogram of all significant correlations (blue, negative; orange, positive) between gene expression and all VMRs and VARs within 2 Mb of the TSS. Bottom, magnified view of the indicated region. **h**, Genes binned according to methylation change downstream of their TSS. Bar colour denotes the proportion of genes upregulated or downregulated, according to their transcript expression in the NSC lineage; parenthesized numbers denote gene number per bin. NS, not significant. **i**,**j**, Overlap of significantly correlating VMRs and VARs with gene features (**i**) and candidate *cis*-regulatory elements (**j**). UTR, untranslated region.

It was long thought that mammalian brains lose the ability to generate new neurons during adulthood. It is now known that adult neurogenesis occurs but is limited to specialized niches including the dentate gyrus and the ventricular–subventricular zone (vSVZ). In the mouse vSVZ, specialized astrocytes that reside in the walls of the lateral ventricles act as adult neural stem cells (NSCs) (Fig. 1a). Upon activation, NSCs become transit-amplifying progenitors (TAPs) that undergo multiple rounds of division and give rise to neuroblasts. Neuroblasts then migrate along the rostral migratory stream (RMS) to the olfactory bulb, where they differentiate into interneurons that integrate into the existing neural circuitry. To a lesser extent, NSCs also give rise to glia, including other types of astrocytes and oligodendrocytes^1–3^.

The advent of single-cell RNA sequencing (scRNA-seq) enabled the characterization of gene expression changes along the neurogenic lineage at unprecedented resolution^4–6^. These studies showed that NSCs can be found in a quiescent or an active state. Quiescent NSCs express genes associated with their astrocyte phenotype, including genes involved in lipid metabolism and glycolysis, which are gradually downregulated during the transition into the active NSC state. Thus, quiescent NSCs have a transcriptome that is no different from that of other astrocytes, including the parenchymal astrocytes of the adjacent striatum and cortex, which are generally considered non-neurogenic. This observation raises hopes for regenerative medicine, which aims to recruit these astrocytes to replace lost neurons. Indeed, several recent in vivo studies have reported astrocyte-to-neuron conversion by ablation or overexpression of key factors in the hippocampus, cortex and striatum^8–11^. Other studies have reported that injury alone is sufficient to induce neurogenesis in some striatal astrocytes^7,10,12^, raising the question of whether all astrocytes have latent neurogenic potential that is merely blocked during homeostasis.

Recently, scRNA-seq methods have been extended to enable simultaneous profiling of gene expression and epigenetic marks. Here we utilize and further develop single-cell nucleosome, methylome and transcriptome sequencing (scNMT-seq^13^), which characterizes three modalities simultaneously for each cell: the transcriptome (cytoplasmic and nuclear mRNA), chromatin accessibility and DNA methylation. This enabled us to assess whether gene expression changes in the NSC lineage are underpinned by epigenetic changes. Furthermore, we compared NSCs (neurogenic vSVZ astrocytes) with non-neurogenic astrocytes from the striatum and cerebral cortex to determine whether NSC stemness is encoded in the epigenome.

## scNMT-seq of the adult NSC lineage

**Extended Data Fig. 1:**
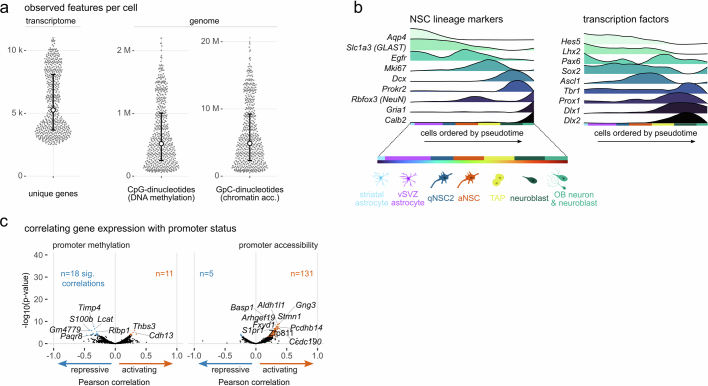
Quality measures and marker expression. **a**, scNMT-seq quality metrics for all cells that passed quality filtering. “Unique genes” refers to the number of genes with at least one sequencing read per cell. “CpG/GpC-dinucleotides” refers to the number of methylation sites with sequencing coverage and thus known methylation status (large circle: median; error bar: interquartile range). For quality metrics of all cells reported in this study, see Supplementary Fig. 1. **b**, Gene expression of key marker genes and transcription factors along pseudotime. **c**, Correlation of promoter methylation (left) and promoter accessibility (right) with gene expression across cells. Negative correlations (blue) indicate a repressive effect of methylation/accessibility; positive correlations (orange) indicate activation. n: number of nominally significant correlations (Pearson’s two-sided correlation test) after Benjamini-Hochberg adjustment; y axis: unadjusted p-values.

We first examined the transcriptome, methylome and chromatin accessibility of NSCs and their progeny. We isolated NSCs and their progeny from the vSVZ via flow cytometry using surface expression of GLAST (also known as SLC1A3), as previously described^14^. Of note, although GLAST transcripts (encoded by *Slc1a3*) are present only in astrocytes and NSCs, the protein is also detected in early neuroblasts, and thus GLAST^+^ sorted cells comprise astrocytes, NSCs, TAPs and early neuroblasts^14^. Furthermore, we isolated astrocytes (GLAST^+^) from the striatum, as well as late neuroblasts (polysialylated neural cell adhesion molecule (PSA-NCAM)^high^) and neurons (PSA-NCAM^low^) from the late RMS and olfactory bulb, as previously described^15^. These cells were analysed by scNMT-seq to quantify gene expression, genome-wide DNA methylation and genome-wide chromatin accessibility at single-cell resolution (Fig. 1b). We increased the throughput^13^ from 96 to 384 cells per run, miniaturized the volume and updated the scRNA-seq protocol to Smart-seq3^16^ (for our modified scNMT-seq protocol see ref. ^17^). After stringent quality filtering and removal of off-target cells, we obtained a total of 540 triple-omic cells with an average of 5,811 detected genes and 678,186 observed methylation sites (genomic CpG dinucleotides) per cell (Extended Data Fig. 1a, Supplementary Fig. 1 and Supplementary Tables 1 and 2).

Integration of our single-cell transcriptomes with a larger scRNA-seq reference dataset of the vSVZ and olfactory bulb^18^ revealed a continuum comprising previously described^14^ sub-groups of dormant NSCs (qNSC1), quiescent NSCs (qNSC2), active NSCs (aNSC), TAPs, neuroblasts and neurons, as well as several oligodendrocytes (Fig. 1c, left). Data from each epigenomic molecular layer alone was also sufficient to distinguish these cell states (Fig. 1c), suggesting that both DNA methylation and chromatin accessibility exhibit dynamic changes along this lineage. We then used Multi-Omics Factor Analysis v2^19^ (MOFA+), a statistical framework for integration of multi-omic single-cell data, to reduce the data to 15 dimensions that incorporate information from all three molecular layers. We used this representation of the data to compute a 2D embedding (uniform manifold approximation and projection (UMAP); Fig. 1d) and to order the cells according to their progression in the NSC lineage (pseudotime). Our cell state assignments and the pseudotime ordering agree with the definitions from the literature as indicated by the expression of common marker genes and known lineage transcription factors (Extended Data Fig. 1b). To assess the quality of our epigenomic data, we next quantified DNA methylation and chromatin accessibility at transcription start sites (TSSs) and CTCF-binding sites in single cells (Supplementary Fig. 2). Figure 1e shows these profiles for a single exemplary neuroblast. As previously reported, the average TSS exhibits low methylation and is accessible^13^. The average CTCF-binding site shows a similar pattern but has more pronounced nucleosome marks^20^ and decreased accessibility where CTCF binds.

## Correlating transcriptome and epigenome

Next, we correlated epigenetic features with gene expression to identify regulatory features that are active in the vSVZ (Fig. 1f–j). Whereas promoter accessibility correlated with gene expression, we found little evidence for dynamic changes in promoter methylation (Extended Data Fig. 1c). Thus, we scanned the entire genome for variably methylated regions^21^ (VMRs) and variably accessible regions (VARs). Unlike VARs, VMRs were more predictive of gene expression than promoter regions and often occurred about 3 kb downstream of the TSS in the first intron (Fig. 1f–j). Indeed, the vast majority of genes that acquired additional methylation downstream of the TSS during NSC lineage progression decreased their transcript expression and vice versa (Fig. 1h). Our findings support the notion that DNA methylation downstream of the TSS^22^ in the first intron^23,24^ silences gene expression and the observation that promoter methylation is less dynamic than methylation at other regulatory elements such as enhancers^25,26^.

## Methylation dynamics of the NSC lineage

**Fig. 2:**
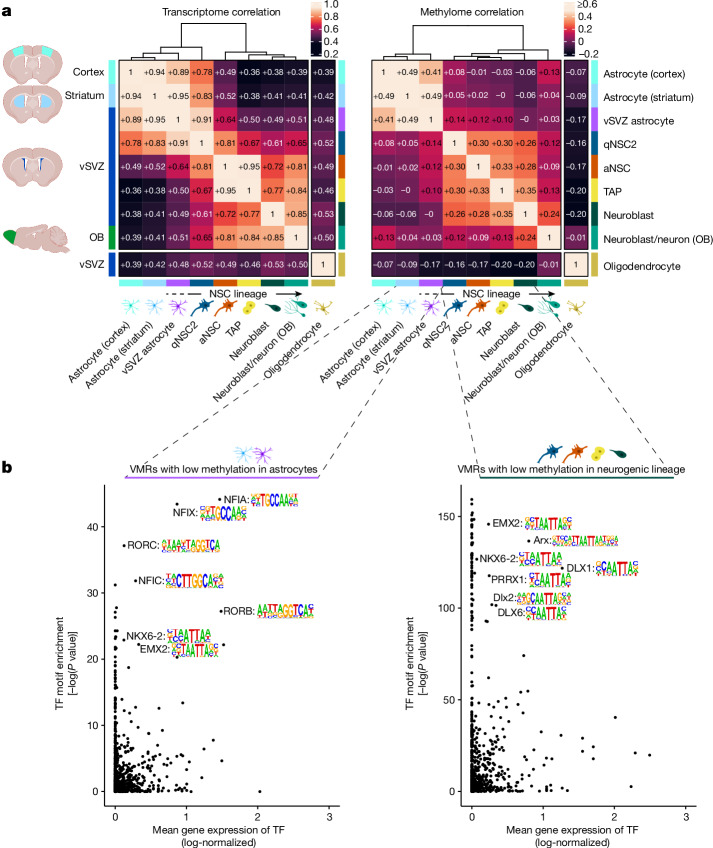
Despite similar gene expression, NSCs possess a unique methylome that distinguishes them from other astrocytes. **a**, Correlation matrices and hierarchical clustering of gene expression and DNA methylation data, averaged for each cell state. Left, correlation of log-normalized expression values for genes expressed in at least 10% of cells. Right, correlation of methylation values for the 40,000 VMRs with the highest sequencing coverage. **b**, Motif enrichment of VMRs with low methylation in astrocytes (vSVZ and striatum) or cells of the neurogenic lineage. The scatter plots of transcription factors (TFs) show the unadjusted one-sided enrichment *P* values reported by HOMER^61^ for the transcription factor motif on the *y* axis, and the mean gene expression for the transcription factor in the respective cell population on the *x* axis. Human transcription factors are set in all upper case and mouse transcription factors are set with initial upper case only.

**Extended Data Fig. 2:**
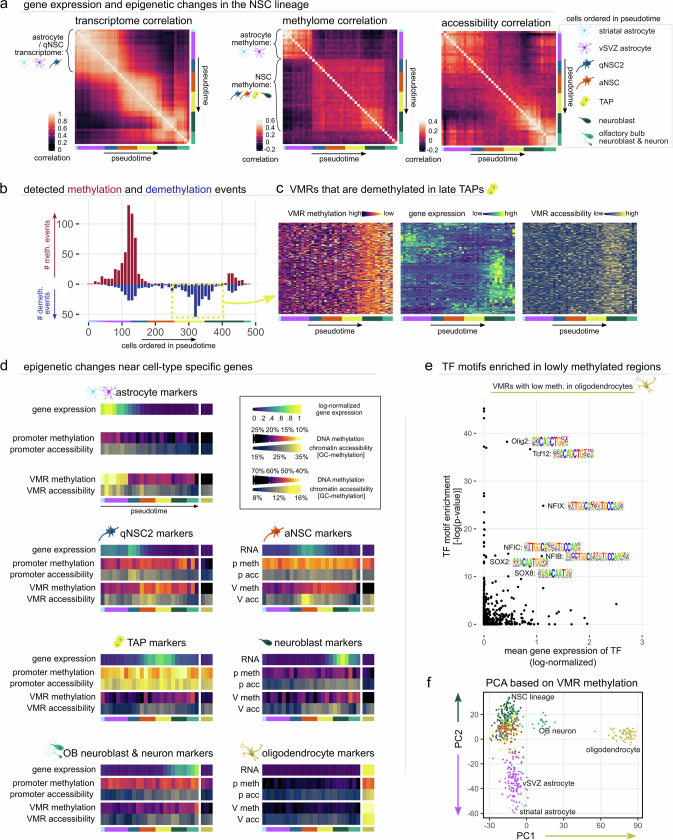
Adult neurogenesis involves a major and a minor wave of methylation change. **a**, Correlation matrices of transcriptomes, methylomes and chromatin accessibility. Cells are ordered and binned according to pseudotime (based on triple-omic MOFA+ factors), with each bin containing 10 cells on average. **b**, Histogram of inferred pseudotime points where VMRs become methylated (red) or demethylated (blue). **c**, Expression and epigenetic state of genes which intersect a VMR that becomes demethylated at the late TAP stage (i.e., the “second wave of demethylation”, dashed orange box in **b**). Rows ordered by hierarchical clustering of gene expression values. Note their expression in neuroblasts. **d**, Expression and epigenetic status of genes expressed at different states of the NSC lineage. The top heatmap row indicates the average expression of 100 marker genes per cell state, identified in a differential expression analysis of the single-cell transcriptomes. Middle and bottom rows indicate the average DNA methylation (meth) and chromatin accessibility (acc) at the 100 promoters (p) of these markers, and at 100 VMRs (V) overlapping marker gene bodies. While gene expression of astrocyte markers fades gradually, the methylation of nearby VMRs is clearly distinct between vSVZ astrocytes and qNSC2. The promoters of TAP markers (cell cycle genes) are demethylated and accessible in all cell states. Oligodendrocyte marker expression coincides with low methylation and increased accessibility of both promoters and VMRs. Markers for intermediate cell states show weaker, more ambiguous epigenetic patterns. **e-f**, Motif enrichment of VMRs with low methylation in oligodendrocytes. **e**, Scatterplot of TFs, showing the TF motif’s unadjusted one-sided enrichment p-values reported by HOMER^61^ on the y axis, and the TF mean gene expression in the respective cell population on the x axis. Human TFs are fully capitalized, the remainder are mouse TFs. **f**, PCA of single-cell methylomes. PC1 and PC2 separate oligodendrocytes and astrocytes, respectively, from the other cells, and informed the regions for motif enrichment in **e** and Fig. 2b.

Several scRNA-seq studies^4–6^ have demonstrated that NSC differentiation is characterized by gene expression changes, but whether this entails changes in DNA methylation has yet to be determined. To quantify the pace of changes along the lineage, we binned cells in pseudotime and calculated, for each of the three modalities, the correlation between the pseudotime bins (Extended Data Fig. 2a). As expected, rapid change in the transcriptomic profile coincides with the activation of NSCs (qNSC2 to aNSC) and with the differentiation of TAPs to neuroblasts. The methylation heat map (Extended Data Fig. 2a, middle), by contrast, shows a markedly different pattern, characterized by a very clear separation of qNSC1 and qNSC2. Of note, the dormant NSC (qNSC1) methylome closely resembles that of striatal astrocytes, which suggests that qNSC1 cells possess the epigenetic makeup of non-neurogenic astrocytes. To test this idea, we acquired scNMT profiles of additional GLAST^+^ cells from the cerebral cortex, striatum and vSVZ. In line with the prevailing view that NSCs are specialized astrocytes^2,27^, hierarchical clustering of transcriptome data grouped qNSC1 and qNSC2 cells with astrocytes from other tissues (Fig. 2a). By stark contrast, methylome clustering grouped only qNSC1 cells with astrocytes of other regions, whereas qNSC2 cells grouped with other cells of the NSC lineage. As qNSC1 cells share their transcriptome and methylome with astrocytes from the striatum and cortex, we hereafter refer to them as vSVZ astrocytes. However, it is important to note that vSVZ astrocytes—also called B1 astrocytes—qualify as stem cells, since they express the stem cell marker proteins TLX and TROY, unlike astrocytes in other areas, express prominin 1 and are thus ciliated, and are able to generate differentiated progeny as shown by lineage tracing studies^5,28,29^. Nevertheless, the most striking readout of our methylome data is the separation between vSVZ astrocytes and qNSC2 that is not apparent in gene expression or chromatin accessibility (Fig. 2a and Extended Data Fig. 2a). Thus, we observed cells with similar transcriptomes and accessibility profiles that are endowed with distinct methylomes. Together, our data suggest that vSVZ astrocytes exhibit an astrocyte methylome that is reprogrammed into an NSC methylome when transitioning to the qNSC2 stage.

Inferring change points by fitting a step function to the methylation values of each VMR across pseudotime revealed a first major wave of both methylation and demethylation in the transition from vSVZ astrocyte to qNSC2, and a second wave of demethylation in late TAPs (Extended Data Fig. 2b). A closer examination of genes affected by demethylation in this second wave indicates that they are predominantly expressed in neuroblasts (Extended Data Fig. 2c), suggesting that demethylation in late TAPs licenses neuroblast genes for later expression. Notably, demethylation of these regions is accompanied by an only transient period of chromatin accessibility. In most cases, accessibility coincides with gene expression, whereas low methylation persists even in those genes that are downregulated at the neuron stage. To assess epigenetic regulation of other state-specific genes, we visualized their average gene expression, promoter methylation and the epigenetic status of nearby VMRs (Extended Data Fig. 2d and Supplementary Table 3). This revealed a sharp increase in methylation near astrocyte markers at the qNSC2 stage, whereas their gene expression fades more gradually. Oligodendrocyte marker expression is clearly underpinned by epigenetic features, whereas TAP markers appear constitutively demethylated and accessible.

Finally, we screened regions that were demethylated specifically in either oligodendrocytes, astrocytes or the neurogenic lineage for enriched transcription factor motifs (Fig. 2b and Extended Data Fig. 2e,f). Regions that are demethylated in astrocytes frequently contain the motif of one or more nuclear factors, including NFIA, which is known to induce demethylation of the astrocyte marker GFAP and is used to convert human induced pluripotent stem cell-derived NSCs to astrocytes^30^, and NFIX, which regulates NSC quiescence and suppresses oligodendrogenesis^31^. Oligodendrocyte-specific regions were enriched for the motifs of OLIG2, a master regulator of oligodendrocyte cell identity^32^, and TCF12, which may be involved in the generation of oligodendrocyte-fated NSCs by Wnt ligands^33^ (Extended Data Fig. 2e). Whether binding of the identified transcription factors is affected by DNA methylation^34^ or whether the transcription factors can affect methylation themselves^30,35,36^ remains to be determined.

## NSCs have a pro-neurogenic methylome

**Fig. 3:**
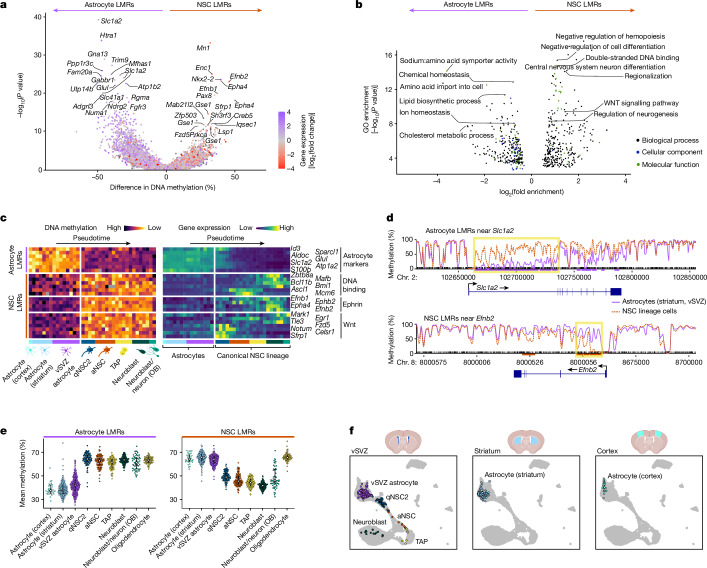
NSCs possess a pro-neurogenic methylome that clearly distinguishes them from common parenchymal astrocytes. **a**, Volcano plot of VMRs, tested for differential methylation between astrocytes (vSVZ and striatum) and cells of the NSC lineage (two-sided Wilcoxon test). VMRs are coloured according to differential expression of the nearest gene. Some genes, such as *Slc1a2*, intersect multiple VMRs. VMRs with Benjamini–Hochberg adjusted *P* value < 0.05 were labelled as LMRs. The *y* axis shows unadjusted *P* values. **b**, GO term enrichment of genes near astrocyte LMRs and NSC LMRs from **a**. The *y* axis shows unadjusted one-sided GREAT^62^ binomial *P* value. **c**, Heat map of methylation level (left) and the expression of intersecting genes (right) for selected LMRs along pseudotime. Rows are ordered by hierarchical clustering of gene expression values. Note the clear separation of astrocytes and cells of the canonical NSC lineage (qNSC2→neuroblast) in the methylation data. **d**, Methylation tracks of *Slc1a2* and *Efnb2*. The curves depict the smoothed average methylation in two pseudobulk cell populations (purple, striatal and vSVZ astrocytes; red, qNSC2→neuroblast). LMRs are marked by red or purple lines below the tracks. *Slc1a2* represents an extreme case with a long stretch of differential methylation, whereas differential methylation at *Efnb2* occurs predominantly at the first intron (both highlighted in yellow). **e**, Mean methylation of astrocyte LMRs and NSC LMRs in *n* = 1,880 cells from 10 samples. Error bars indicate interquartile range; white dots show the median. **f**, Independent sample of GLAST^+^ cells from three tissues, used to assess astrocyte LMR methylation in the cortex. scNMT-seq transcriptomes are integrated with data from ref. ^18^, corresponding methylomes represent subset of the cells in **e**.

We then focused on addressing whether the substantial differences in DNA methylation between vSVZ astrocytes and qNSC2 account for their distinct functions. Detection of differentially methylated regions (DMRs) identified low-methylation regions (LMRs) in astrocytes (vSVZ, striatum) and qNSC2, and many genes near these were more highly expressed in the respective subtype (Fig. 3a and Supplementary Table 4). LMRs in striatal and vSVZ astrocytes occur near genes involved in transport and metabolism of amino acids (*Slc1a2* and *Glul*), ions (*Slc41a2*) and cholesterol (*Lcat*), among others (Fig. 3b). Since these Gene Ontology (GO) terms represent fundamental astrocyte functions, we labelled these loci ‘astrocyte LMRs’, reasoning that their demethylation might be associated with an astrocyte cell identity. To test this hypothesis and assess the reproducibility of our findings in other brain areas, we quantified astrocyte LMR methylation in our independent sample of astrocytes from the cerebral cortex (Fig. 3e,f). In line with our expectations, our astrocyte LMRs exhibited low methylation in all common parenchymal astrocytes regardless of their tissue of origin (vSVZ, striatum or cortex), but not in other glial cells (oligodendrocytes). Of note, vSVZ astrocytes showed slightly higher average levels of methylation in astrocyte LMRs than other astrocytes. This suggests that vSVZ astrocytes might be epigenetically closer to NSCs than other astrocytes, perhaps owing to the presence of pro-neurogenic factors in this niche.

**Extended Data Fig. 3:**
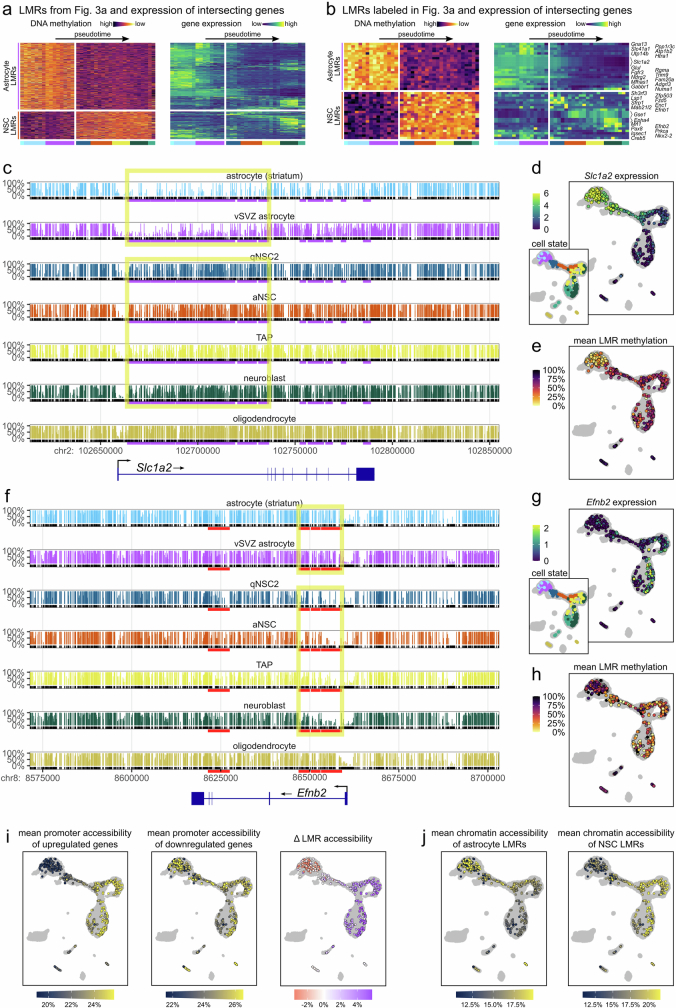
Detailed view of astrocyte LMRs, NSC LMRs and expression of intersecting genes. **a-b**, Heatmaps of LMR methylation (left) and expression of intersecting genes (right) in pseudotime. **a**, All significant LMRs which intersect a gene. **b**, Labeled LMRs from Fig. 3a. Note that some genes intersect multiple LMRs. Rows are ordered by hierarchical clustering of gene expression values. Cells are separated by tissue and then binned in pseudotime. **c**, Detailed view of DNA methylation near *Slc1a2* in pseudobulk samples of each cell state. Each CpG is represented by a colored vertical line representing its average methylation value in the pseudobulk sample. Short black lines denote the position of CpGs with sequencing coverage. Colored purple/red bars denote LMRs. **d-e**, Log-normalized expression of *Slc1a2* and mean methylation of LMRs intersecting this gene. **f-h**, Same visualization for *Efnb2*. For expression and methylation of additional NSC / astrocyte markers, see Supplementary Figs. 4–6. **i-j**, Chromatin accessibility of naive wild-type cells. **i**, Average promoter (TSS ± 1 kb) accessibility of genes up- or downregulated in the NSC lineage. To minimize the impact of outliers on the color scale, the 1% (methylation) or 5% (accessibility) highest and lowest values are clipped. **j**, Mean chromatin accessibility of astrocyte LMRs and NSC LMRs, as well as the difference between these two means (bottom, analogous to the methylome score).

By contrast, genes near LMRs in qNSC2 are enriched for regulators of cell differentiation (*Ascl1* and *Dlx1*) and DNA binding (*Nkx2-2* and *Pax8*) (Fig. 3a–c and Extended Data Fig. 3a,b). Among the top hits are several genes associated with the ephrin signalling pathway (*Efnb2*, *Epha4* and *Efnb1*), which controls neuroblast migration and NSC quiescence^2,37–39^. This implies that demethylation at these regions contributes to stem cell function. Thus, we labelled these regions ‘NSC LMRs’. We conclude that astrocytes and qNSC2 cells, two populations that are difficult to distinguish by their transcriptome^2,27^, exhibit very distinct methylomes, tied to either stem cell function in the case of qNSC2, or to astrocyte function in the case of striatal, cortical and vSVZ astrocytes. This clear divide does not translate to the transcriptome, however: the excitatory amino acid transporter gene *Slc1a2*, for instance, exhibits low methylation in astrocytes of the vSVZ, striatum and cortex, but is highly methylated at qNSC2 and later stages of the NSC lineage (Fig. 3d). Notably, this gene is transcribed up until the aNSC stage, and its expression subsequently decreases gradually (Extended Data Fig. 3c–e). The residual presence of astrocytic transcripts in qNSC2 despite methylation might be due to the transition from vSVZ astrocytes to qNSC2 without cycling and thus without dilution of these transcripts.

Genes near NSC LMRs show highest expression at different points of the NSC lineage, although methylation is low at all stages (Fig. 3c and Extended Data Fig. 3a,b). This indicates that neurogenic genes are already epigenetically licensed at the qNSC2 stage for later expression. Among these genes are ephrin signalling genes expressed in neuroblasts (for example, *Efnb2*; Extended Data Fig. 3f–h), genes encoding DNA-binding proteins expressed after NSC activation, and Wnt signalling genes, of which some are specifically expressed at the qNSC2 stage (Fig. 3c).

Next, we explored whether the epigenetic divide between NSCs and common parenchymal astrocytes (of the vSVZ, striatum and cortex) is visible in other epigenetic features. To investigate this, we quantified promoter chromatin accessibility of genes that are either transcriptionally upregulated or downregulated in the NSC lineage compared with striatal or vSVZ astrocytes (Extended Data Fig. 3i). Although the average upregulated gene promoter was indeed more accessible in the NSC lineage, we did not observe an abrupt divide between NSCs and astrocytes, but rather a gradual increase during the course of NSC activation. A similar pattern was observed when quantifying chromatin accessibility of astrocyte and NSC LMRs, which mirrored the methylation state of these regions but did not recapitulate the strong divide observed in the methylome (Extended Data Fig. 3j).

To summarize, we found vast methylation differences between cells endowed with stemness and common parenchymal astrocytes of the striatum, cortex and vSVZ, despite their remarkably similar transcriptomes. We propose that it is DNA methylation that establishes the difference between stemness and supporting parenchymal astrocyte function.

## Methylome remodelling upon ischaemia

**Fig. 4:**
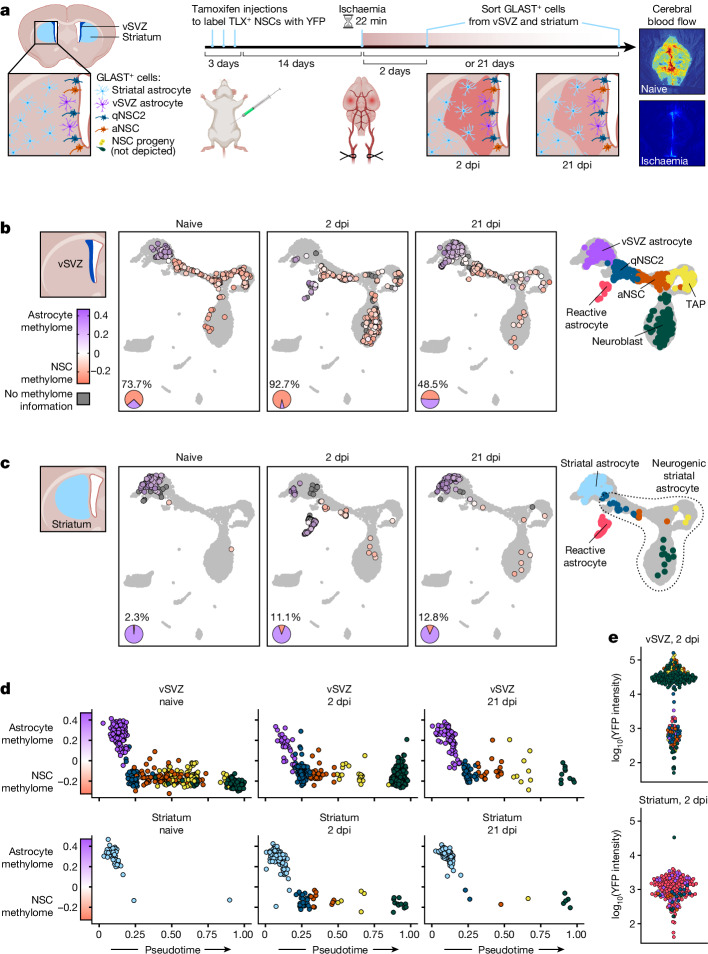
Ischaemic injury induces an NSC methylome in striatal astrocytes. **a**, Experiment to assess the effects of ischaemic injury on GLAST^+^ cells in the vSVZ (astrocytes, NSCs and NSC progeny) and striatum (astrocytes). Both tissues were analysed by scNMT-seq at 2 dpi and 21 dpi. Tamoxifen injections label TLX^+^ NSCs via Cre-inducible YFP expression to detect potential NSCs that may migrate to the striatum. Right, laser speckle imaging of cerebral blood flow in representative naive and ischaemic mouse brains. **b**, UMAP visualization of GLAST^+^ triple-omic cells isolated from the vSVZ (large points), integrated with a larger scRNA-seq dataset^18^ (grey shaded region). UMAP coordinates reflect the transcriptomic state and colour reflects the methylome state (Extended Data Fig. 5a,b) of each cell. Pie charts show the proportion of cells inside the neurogenic lineage (qNSC2→neuroblast lineage is shown in salmon). **c**, GLAST^+^ cells isolated from the striatum, depicted as in **b**. **d**, Methylome state of cells in transcriptome-based pseudotime. **e**, TLX–YFP fluorescence intensity for a subset of cells that were index-sorted, demonstrating that cells in the striatum do not derive from TLX^+^ vSVZ NSCs.

**Extended Data Fig. 4:**
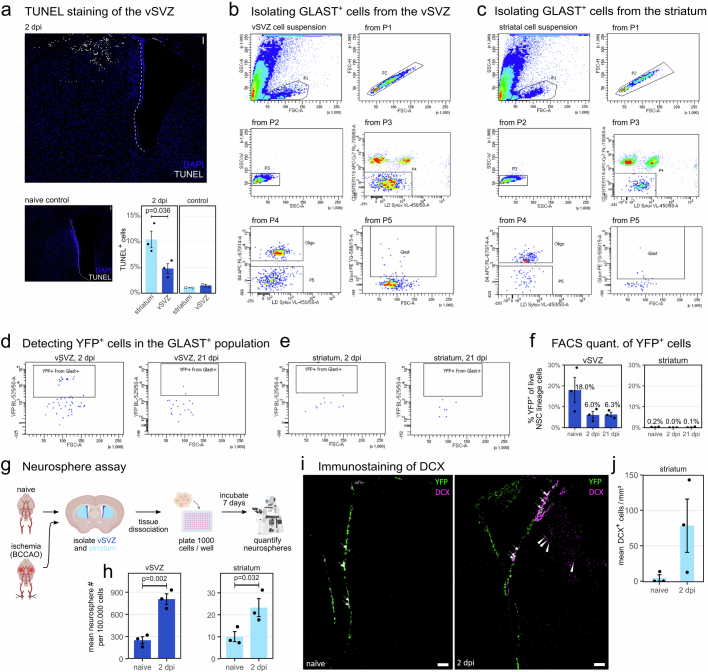
Cell sorting demonstrates that cells captured from the striatum did not migrate from the vSVZ. **a**, Representative confocal images of the SVZ and striatum from a naive and a post-ischemic (2 dpi) mouse brain stained with TUNEL (terminal deoxynucleotidyl transferase-dUTP nick end labeling; labels apoptotic cells^86^). Brain slides are counterstained with DAPI (blue). TUNEL^+^ nuclei (segmented with Cellpose^68^) are shown in white. Scale bar: 100 µm. Bottom right: Quantification of TUNEL^+^ cells in the vSVZ and striatum of n = 5 mice depicted with 5 shapes (solid shapes: ischemic mice; outlined triangle: naive control; outlined square: sham-operated control; one-sided paired t test). **b-c**, FACS Strategy for sorting GLAST ^+^ cells from the vSVZ (**b**) and striatum (**c**). Striatal GLAST ^+^ cells correspond to astrocytes, while vSVZ GLAST ^+^ cells correspond to NSCs / vSVZ astrocytes. Dead cells and CD45/O4/Ter119 positive cells were excluded. **d-e**, The YFP intensity of GLAST ^+^ cells was captured by index sorting. Dot plots show the fraction of YFP^+^ cells from the GLAST ^+^ population, being higher in the vSVZ sample 2 dpi. Antigen names are written on each axis, followed by the filter used to measure the signal. FACS: Fluorescence-activated cell sorting. SSC-A: Side scatter area. FSC-A: forward scatter area. FSC-H: forward scatter height. RL-780/60-A: APC-Cy7 fluorochrome. VL-450/50-A: Pacific blue fluorochrome. RL-670/14-A: APC fluorochrome. YG-586/15-A: PE fluorochrome. **f**, FACS quantification of cells of the naive and post-ischemic vSVZ and striatum (n = 3 individual mice per condition). Shown is the percentage of YFP^+^ (TLX reporter) cells among astrocytes and NSC lineage cells after TAM injection. **g-h**, Neurosphere assay of cells isolated from the naive and post-ischemic vSVZ and striatum (n = 3 individual mice), quantified in **h**. Welch’s two-sided t-test. **i-j**, Immunofluorescence staining of DCX in the naive and post-ischemic vSVZ and striatum of TiCY mice, showing absence of YFP^+^DCX^+^ cells in the striatum. Arrows: YFP^+^DCX^+^ neuroblasts in the vSVZ; arrowheads: YFP-DCX^+^ neuroblasts in the striatum. **j**, Quantification of DCX^+^ cells in the striata of n = 6 individual mice (points). No YFP^+^DCX^+^ cells were observed. Bars and error bars in panels **a**, **f**, **h**, **j** represent mean and standard error.

Next, we aimed to test whether the unique methylation profile that we observed in NSCs supports stemness. Ideally, an NSC methylome would be induced in common parenchymal astrocytes to observe whether these cells gain the ability to produce neurons. Although recent advances in CRISPR–Cas9-based technologies enable precise epigenome editing^40,41^, targeting hundreds of LMRs in vivo remains unfeasible. Instead, we tested whether gain of neurogenic capabilities entails gain of an NSC methylome. Previous studies demonstrated that the production of neuroblasts can be increased by an ischaemic injury to the brain. In the vSVZ, ischaemia activates vSVZ astrocytes to exit their quiescent state in an interferon-dependent manner^5^. Similarly, ischaemic injury triggers a neurogenic programme in astrocytes of the striatum, leading to the production of neuroblasts^7,12^. To induce gain of neurogenic capabilities in astrocytes and observe potential effects on the methylome, we subjected 2-month-old mice to transient global brain ischaemia for 22 min, which leads to death of medium spiny neurons and white matter damage in the striatum, but not in the vSVZ^42^ (Extended Data Fig. 4a and Supplementary Fig. 3). Two days post-ischaemia (dpi) and at 21 dpi, we isolated GLAST^+^ cells^14^ from the vSVZ and striatum for scNMT-seq (Fig. 4a), yielding triple-omic profiles for 809 cells (Supplementary Table 1). As a point of reference, we also sequenced additional GLAST^+^ astrocytes from the naive striatum. To ensure that isolated striatal cells were not contaminated with cells arising from the vSVZ, we labelled TLX^+^ vSVZ NSCs^43^ two weeks prior to ischaemia as in ref. ^29^. Reassuringly, we detected YFP^+^GLAST^+^ cells only in the vSVZ and not in the striatum (Fig. 4e and Extended Data Fig. 4b–f). To confirm that our injury model elicits a neurogenic response outside the neurogenic niche as reported previously^7,10,12^, we performed neurosphere assays on the vSVZ and striatum isolated from naive and post-ischaemic mice, confirming increased neurosphere formation in both tissues after ischaemia (Extended Data Fig. 4g,h). Immunofluorescence staining further revealed the emergence of cells expressing the neuronal migration protein doublecortin (DCX) upon ischaemia (Extended Data Fig. 4i,j). Notably, none of the DCX^+^ cells expressed YFP, demonstrating that they were not derived from TLX^+^ vSVZ NSCs.

Integrating the transcriptomes of GLAST^+^ cells from naive, 2 dpi and 21 dpi mice with the larger scRNA-seq dataset^18^ revealed that in contrast to naive conditions, which involve a very low activation rate of dormant vSVZ astrocytes^44^, ischaemia triggers the majority of vSVZ astrocytes to transition into the qNSC2 state (Fig. 4b), and thereby become depleted. This confirms the previously described activation of neurogenesis in dormant vSVZ astrocytes^5^. Similarly, our data show that injury also activates this neurogenic programme in striatal astrocytes, as previously reported^7,10,12^. Whereas striatal astrocytes exhibit a uniform transcriptome under naive conditions, this population is depleted at 2 dpi and instead we observe astrocytes that are transcriptionally similar to qNSC2 cells (Fig. 4c) and are therefore neurogenic, as also evidenced by our observation of cells resembling TAPs and neuroblasts at 2 dpi. Of note, these neuroblasts retain protein expression of GLAST (and mRNA expression of other astrocyte markers, Supplementary Fig. 4) at later stages, which indicates faster differentiation following ischaemia (recall that in Fig. 4, we only show GLAST^+^ cells, whereas Fig. 1 also includes—for example, PSA-NCAM^+^ neuroblasts).

**Extended Data Fig. 5:**
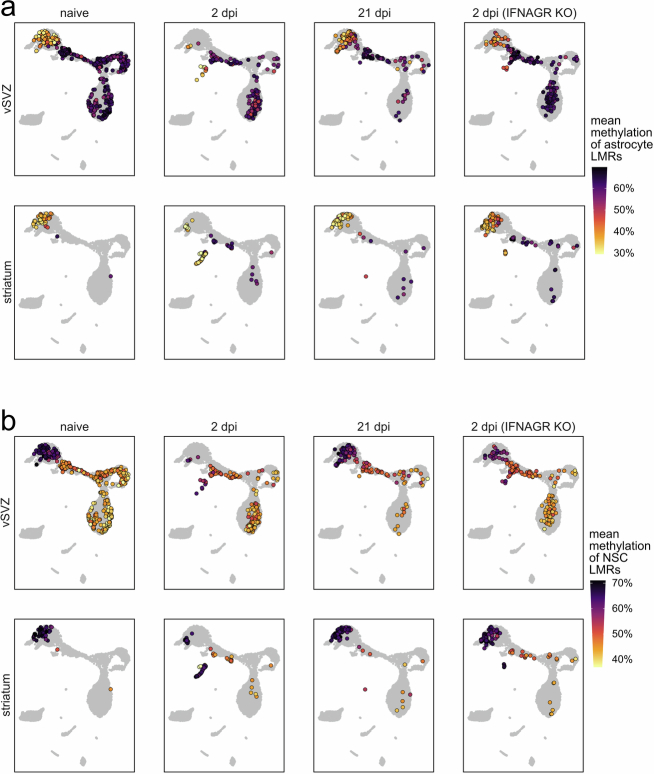
Methylation and chromatin accessibility of astrocyte LMRs and NSC LMRs. **a**-**b**, Average methylation of astrocyte LMRs (**a**), and NSC LMRs (**b**) from Fig. 3. The methylome score introduced in Fig. 4b is the difference between these two averages.

Most importantly, in the vSVZ and striatum, ischaemia-induced neurogenic astrocytes do not display an astrocyte methylome, characterized by low methylation at astrocyte LMRs and high methylation at NSC LMRs. Instead, these cells exhibit an NSC methylome, with high methylation of astrocyte LMRs and low methylation of NSC LMRs (Fig. 4b–d and Extended Data Fig. 5a,b). This demonstrates that ischaemia-induced gain of neurogenic capabilities is accompanied by substantial epigenetic remodelling, including demethylation of neuroblast-specific genes. Overall, this finding strengthens our view that neurogenesis and stem cell function requires an NSC methylome. This process is already reversed at 21 dpi—at this point, cells with the transcriptome and methylome of naive astrocytes are restored in the vSVZ and striatum (Fig. 4b,c). We also observed a small number of post-ischaemic vSVZ cells with the transcriptional profile of NSC-lineage cells that nonetheless displayed an astrocyte methylome (Fig. 4d). These cells might represent cells transitioning into stemness, which would indicate that as opposed to homeostatic conditions, ischaemia-induced changes in gene expression may precede epigenome remodelling.

**Extended Data Fig. 6:**
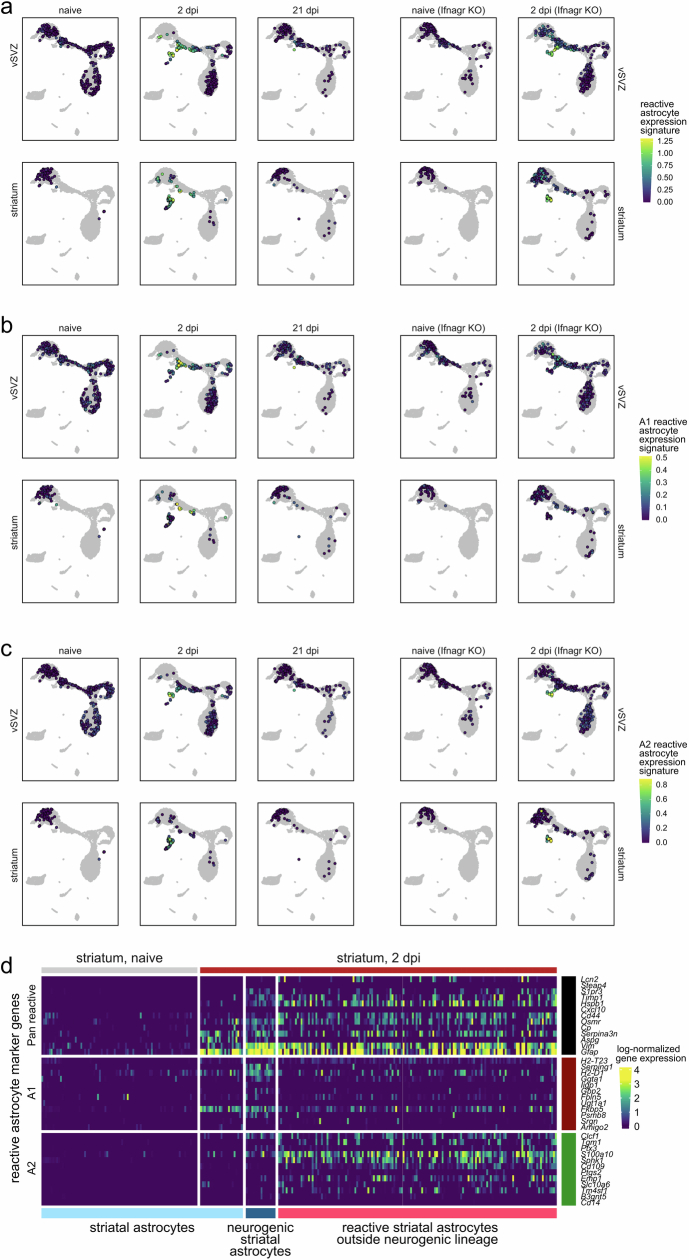
Expression of reactive astrocyte marker genes. **a-c**, Mean log-normalized expression of reactive astrocyte marker genes from^46^. These markers include putative pan reactive marker genes (**a**) and markers observed in two astrocyte subtypes (**b**: A1 reactive astrocytes, **c**: A2 reactive astrocytes). **d**, Heatmap of reactive astrocyte marker gene expression in the naive and post-ischemic striatum.

**Extended Data Fig. 7:**
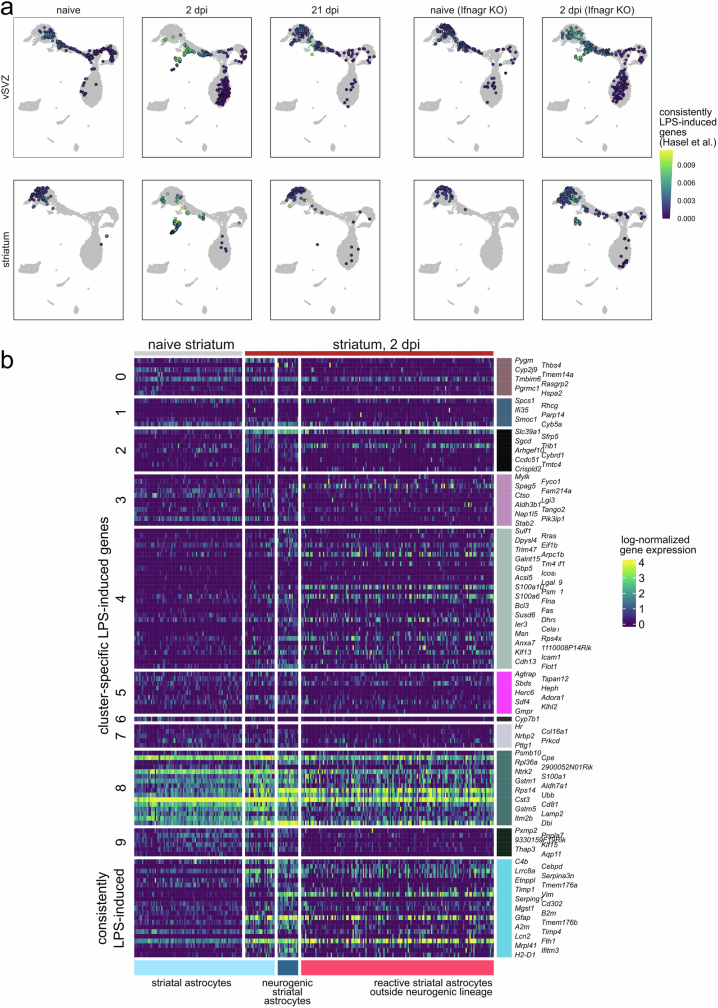
Expression of LPS-induced genes reported by Hasel et al.^47^. **a**, Mean log-normalized expression of genes consistently induced by LPS-injection (from^47^). **b**, Heatmap of LPS-induced genes that were only observed in one of 10 astrocyte sub-types (top) or the consistent genes from **a** (bottom). LPS: lipopolysaccharide.

In addition to increased neurogenesis, samples isolated at 2 dpi also contained a new cell cluster located in transcriptome space outside the neurogenic lineage (Fig. 4b,c). We suspected that these cells might be reactive astrocytes—that is, astrocytes that undergo pronounced changes in function, morphology and gene expression in response to pathology^45^. Indeed, universal reactive astrocyte marker genes^46^^,^^47^ were upregulated at 2 dpi (Extended Data Figs. 6a and 7a). Expression changes during reactive astrogliosis are highly heterogeneous and depend on various factors including the CNS region and the type of pathology^45^. Accordingly, reactive astrocytes in our data did not fall clearly into previously described sub-groups of reactive astrocytes (Extended Data Figs. 6b–d and 7b). Nonetheless, we observed that astrocytes that entered the neurogenic lineage expressed markers associated with the phenotype labelled A1^46^, whereas cells outside the lineage expressed markers observed in the A2 phenotype.

**Extended Data Fig. 8:**
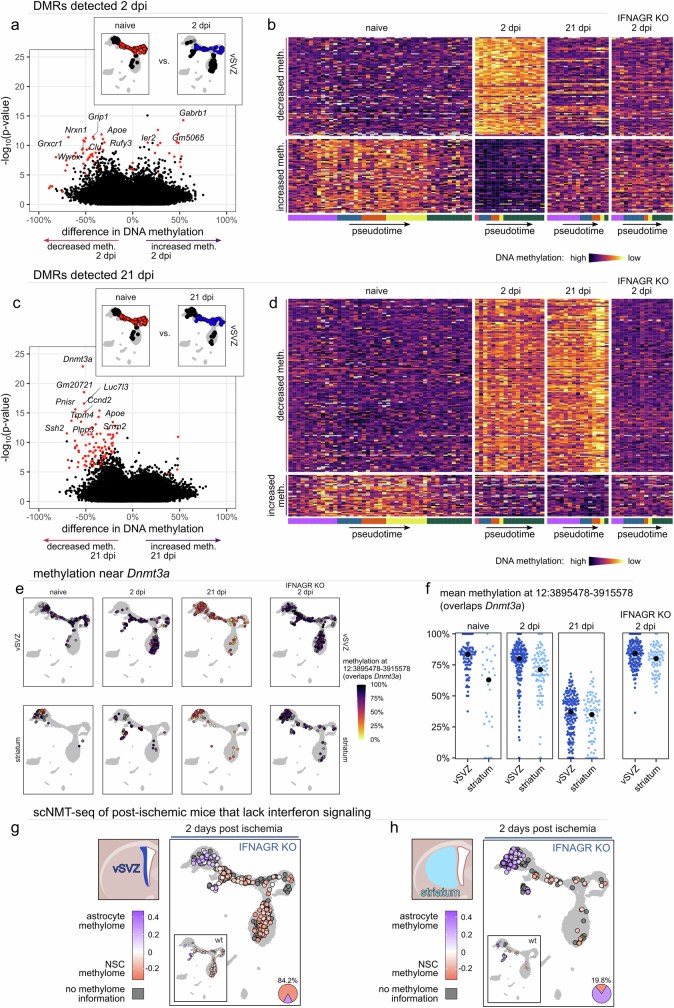
scNMT-seq of post-ischemic mice lacking interferon receptors and DMRs detected after ischemia. **a**, Volcano plot of DMRs detected in wild-type mice 2 dpi. Inset: NSC-lineage cells 2 dpi (blue) were tested against naive (red). y axis: unadjusted two-sided p-value obtained with ‘methscan diff’^21^; red: Benjamini-Hochberg adjusted p < 0.05). **b**, Heatmap of DMR methylation at three timepoints and in post-ischemic IFNAGRKO. **c-d**, Volcano plot and heatmap of DMRs detected in wild-type mice 21 dpi (statistics as in **a**). **e**, Mean methylation of the top DMR from **c**, which overlaps *Dnmt3a*. Cells with no sequencing coverage at this DMR are gray. **f**, Bee swarm plot of data from **e**. Points: single cells, large point: median. **g-h**, GLAST^+^ cells isolated from the IFNAGRKO vSVZ (**g**) or striatum (**h**) 2 dpi, depicted as in Fig. 4b,c. For comparison, the inset depicts GLAST^+^ cells from wild-type 2 dpi. The naive IFNAGRKO control is shown in Extended Data Fig. 6a. Source Data

Our data show that ischaemia is associated with the gain of an NSC methylome. To assess whether ischaemia might also lead to other methylation changes—that is, changes outside of astrocyte LMRs or NSC LMRs detected in naive brains—we searched for potential DMRs between naive and post-ischaemic cells of the neurogenic lineage (Extended Data Fig. 8a–f). At 2 dpi, we detected a modest number of DMRs with both increased and decreased methylation levels. By contrast, ischaemia-specific DMRs detected at 21 dpi were predominantly demethylated, and often already showed decreased methylation levels at 2 dpi. Of note, the most significant DMR at 21 dpi overlaps *Dnmt3a*, which encodes the methyltransferase responsible for de novo DNA methylation (Extended Data Fig. 8c,e,f). Given the surprisingly short timespan during which ischaemia-induced methylation changes are reverted (Fig. 4b,c), this suggests a potential negative feedback loop in which ischaemia first induces demethylation, including at *Dnmt3a*, which is then activated and reverts the ischaemia-induced demethylation events.

Previous studies have identified interferons as potential upstream regulators of injury-induced reactive astrogliosis^47^ and neurogenesis^5,48,49^. Consequently, scNMT-seq of GLAST^+^ cells isolated from post-ischaemic mice that lack receptors for IFNα, IFNβ and IFNγ (IFNAGRKO mice) showed an increased number of vSVZ astrocytes that did not respond to ischaemia (Extended Data Fig. 8g,h, IFNAGRKO cells also shown in Extended Data Figs. 5–7). As expected, cells that successfully entered the neurogenic lineage despite ablation of the interferon receptors possessed an NSC methylome. However, other ischaemia-specific DMRs were not observed in IFNAGRKO cells, suggesting that interferon signalling may be required for their establishment (Extended Data Fig. 8b,d–f).

## *Dnmt3a* and stemness

**Fig. 5:**
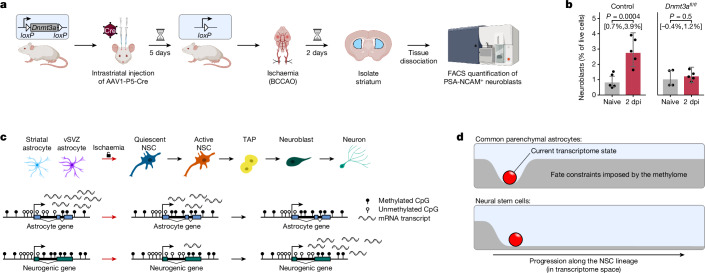
Ischaemic injury does not induce neurogenesis in *Dnmt3a*-deficient mice. **a**, Experiment to assess whether injury-induced neurogenesis in the striatum depends on the de novo DNA methyltransferase DNMT3A. **b**, FACS quantification of PSA-NCAM^+^ neuroblasts in the naive or post-ischaemic striatum of *n* = 9 *Dnmt3a*-deficient mice and *n* = 10 wild-type control mice. Dots represent individual mice, columns represent the mean (on the logit scale) and error bars mark 95% confidence intervals. Brackets mark comparisons, with *P* value for the (two-sided) contrast indicated above and 95% confidence interval for the difference below the bracket. All inference is performed using a linear model fitted on the logit scale. The *P* value for the interaction (that is, against the null hypothesis of the two bracket-marked differences not being equal) is 0.017. **c**, Schematic depiction of genes involved in astrocyte function and neurogenesis, and their methylation and gene expression status in different cell states. **d**, We propose that DNA methylation locks common parenchymal astrocytes in their astrocyte fate by repressing genes required for neurogenesis. By contrast, these genes are demethylated in NSCs, which permits their progression along the neurogenic lineage. Source Data

Our previous experiments showed that striatal astrocytes which enter the neurogenic lineage upon ischaemia acquire an NSC methylome. However, it is not yet clear whether methylome remodelling is strictly required for activation of the stemness programme in astrocytes. To investigate this possibility, we obtained *Dnmt3a*^*fl/fl*^ mice, which enable targeted knockout of *Dnmt3a* by injection of adeno-associated virus carrying the Cre recombinase gene (AAV-Cre), thereby enabling us to impair de novo DNA methylation specifically in the striatum (Fig. 5a). We subjected both *Dnmt3a*-deficient and wild-type control mice to transient ischaemia and assessed neurogenic potential in the striatum by fluorescence-activated cell sorting (FACS) quantification of PSA-NCAM^+^ neuroblasts. As expected, control mice showed a marked increase in the number of detected neuroblasts upon ischaemia (*P* = 0.0004; linear model on logit scale) (Fig. 5b). However, this response is not merely strongly reduced in *Dnmt3a*-deficient mice (*P* = 0.017 for interaction) but seems to be completely absent (effect below 1.2 percent points at 95% confidence level). This suggests that methylome remodelling via DNMT3A is required to acquire neurogenic potential.

## Discussion

The past few decades have demonstrated that new leaps in our understanding of DNA methylation are often driven by technological innovations such as bisulfite sequencing^25,50^. Similarly, our in vivo assessment of DNA methylation in the adult NSC lineage was only possible as a result of recent advances in single-cell multi-omics. This technology enabled us to study defined cell populations without the need for good FACS surface markers or cell culture systems, which are known to alter DNA methylation^51,52^. Classically, DNA methylation was viewed as a repressive epigenetic mark that remained static once established^25^. In recent years, however, this view has been overturned by studies demonstrating that DNA methylation is dynamic in embryonic development^36,53,54^ and in stem cells differentiating in vitro^55,56^. Here we demonstrate that DNA methylation is similarly dynamic in NSCs that are differentiating in adult brains. Our results also suggest that methylation changes in response to environmental stimuli such as ischaemia, indicating that DNA methylation is even more dynamic and that it may have a role in a wide range of biological processes in adult tissues. Our finding that *Dnmt3a* itself is demethylated at 21 dpi suggests that activity of the DNA methylation machinery itself might be regulated by methylation change. For instance, injury-induced methylome remodelling results in demethylation at *Dnmt3a*, which may ultimately lead to altered DNMT3A activity that facilitates the reversal of the injury-induced methylation change in some cells. Further investigations are warranted to unravel such potential feedback loops.

Here we take a crucial step towards understanding why cells with similar transcriptomes can be endowed with either parenchymal supporting functions or stemness. We observe that NSCs of the vSVZ possess a unique DNA methylome, which sets them apart from common parenchymal astrocytes of the cortex and striatum (Fig. 5c). We propose that common astrocytes do not usually give rise to neurons because their methylome locks them in their astrocyte fate by stabilizing the expression of astrocyte genes and by silencing neurogenic genes (Fig. 5d). Thus, the DNA methylome is not only associated with the current transcriptome state of a cell, but may also serve as a blueprint for future transcriptomic states. The putative fate lock imposed by the astrocyte methylome is released upon ischaemic injury, which enables the generation of neuroblasts. The striatum lacks a neurogenic injury response in the absence of the de novo methyltransferase *Dnmt3a*, suggesting that epigenome remodelling is not merely coincidental, but rather has a crucial role. However, it remains to be seen whether changes in DNA methylation are a driving force behind differentiation or whether the methylome is merely a stabilizing constraint on a particular differentiated fate. Targeted manipulation of DNA methylation, for instance using endonuclease dead Cas9 (dCas9)-based epigenome modifiers^40,41^, could be used to answer this question. Related to this, is the question of whether the current strategy of reprogramming through forced expression of transcription factors similarly remodels the methylome, or whether there are additional factors that would enhance the efficiency of reprogramming.

Finally, given the substantial number of epigenetic changes that occur upon ischaemia, this innate astrocyte-to-neuron differentiation may resemble in vitro reprogramming experiments such as astrocyte-to-neuron^57^ or induced pluripotent stem cell-to-neuron conversion^58,59^ via transcription factors. If so, it may be beneficial for reprogramming experiments to consider addressing the methylome to achieve stable and precise cell fates.

This work shows that astrocytes acquire stem cell function through changes in DNA methylation. These changes occur in the neurogenic vSVZ of the adult brain but can also be triggered by acute injury in common striatal astrocytes. Notably, astrocyte and NSC LMRs have been found in human brain tumours^60^. Targeting DNA methylation^40,41^ to gain stemness or astrocyte features offers a potential therapeutic avenue to repair the diseased nervous system or fight cancer^60^.

## Methods

### Animals

The following mouse lines were used: C57BL/6N (for wild-type control, naive condition and neurosphere assay), *Ifnar*^*−/−*^*Ifngr*^*−/−*^ (for IFNAGRKO naive condition) (B6.Cg-*Ifnar1*^*tm1Agt*^ *Ifngr1*^*tm1Agt/Atp*^)^63^, TiCY (B6-Tg(*Nr2e1*-*creERT2*)1Gsc *Gt(ROSA)26Sor*^*tm1(EYFP)Cos*^*Fastm1*^*Cgn/Amv*^, WT-TiCY for wild-type ischaemia condition)^43^, TiCY-IFN(A/G)R-KO (TiCY-IFN-KO, for IFNAGRKO ischaemia condition) (B6-Tg(*Nr2el-CreERT2*)lGsc *Gt(ROSA)26Sor*^*tml(EYFP)Cos*^*Fastm1*^*Cgn*^ *Ifnar1*^*tmlAgt*^ *Ifngr1*^tmlAgt/Amv^)^63,64^, B6 *Dnmt3a* floxed × VE-Cad CreERT2/4 (*Dnmt3a*-flox, for AAV_Cre injection experiment)^65,66^, and TCF-Lef [B6-Tg(TCF/Lef1-HIST1H2BB/EGFP)61Hadj] (WT–TCF-Lef, for cortical and striatal astrocytes)^67^. Mice were male and were age-matched to 2 months old, except for the ‘ischaemia 3 weeks’ mice (3 months old), the TCF-Lef mice (4 months old) and the *Dnmt3a*-flox (2–6 months old). No randomization or blinding was performed; see Supplementary Table 2 for a detailed list of experimental conditions and mouse lines. Animals were housed in the animal facilities of the German Cancer Research Center (DKFZ) at a 12 h dark/light cycle with free access to food and water. Humidity was kept at 55% and temperature at 22 °C. All animal experiments were performed in accordance with the institutional guidelines of the DKFZ and were approved by the Regierungspräsidium Karlsruhe, Germany.

### Tamoxifen injection and ischaemia

Two-month-old TiCY mice were intraperitoneally injected with tamoxifen. In these mice, tamoxifen-induced Cre recombination takes place in NSCs in the vSVZ, which express TLX (*Nr2e1*)^43^, and will stably activate the production of enhanced YFP, labelling NSCs and their progeny. Tamoxifen injection was done as described before^18^. Two weeks after injection, a bilateral common carotid artery occlusion (BCCAO) injury was performed as described^5^. Naive mice or injured mice were euthanized at 2 or 21 dpi for single-cell sorting and sequencing, FACS analysis and immunohistochemistry.

### Single-cell suspension and FACS

For the ischaemia experiment, the dorsolateral walls of the lateral ventricles (vSVZ) and the striatum were isolated. For the naive experiments, vSVZ, striatum, and olfactory bulb were isolated. Depending on the plate, individual or pooled mice were used to sort single cells on plate. For more information see Supplementary Table 2. Tissues were processed as described previously^29^ and sorted in a BD FACSAria or FACSFusion at the DKFZ Flow Cytometry Facility. Cells were stained with the following antibodies (all conditions and tissues together): O4-APC and O4-APC-Vio770 (Miltenyi; diluted 1:100), Ter119-APC-Cy7 (Biolegend; 1:100), CD45-APC-Cy7 (BD; 1:200), GLAST (ACSA-1)-PE (Miltenyi:1:50), PSA-NCAM-PE-Vio770 (Miltenyi; 1:75), Prominin 1-A488 (eBioscience; 1:75), and Sytox Blue (Life Technologies, 1:500). For sorting, we size-selected the vSVZ, striatum, or olfactory bulb cells and excluded for doublets, dead cells and CD45^+^/Ter119^+^ cells as recently described^14^. We then sorted different cell populations according to the tissue and experimental condition as follows (also detailed in Supplementary Table 2): For comparison between naive and post-ischaemic conditions: in the vSVZ we sorted GLAST^+^ cells and O4^+^ cells, in the striatum we sorted GLAST^+^ cells. In the olfactory bulb we sorted PSA-NCAM low and high cells. For the ischaemia experiment, we additionally recorded the YFP information for some samples via index sorting. For determining the signal of YFP in the striatum, we specifically performed FACS quantification of naive and ischaemic TiCY mice (2 dpi and 21 dpi). YFP signal was measured from lineage cells in the vSVZ and striatum. Single cells were sorted into individual wells of a 384-well plate. For a small number of plates, we also sorted two different populations of vSVZ astrocytes/NSCs (GLAST^+^/PROM1^+^ and GLAST^+^/PROM1^−^), PSA-NCAM^+^ neuroblasts and O4^+^ oligodendrocytes. For a detailed breakdown of the populations that were sorted, see Supplementary Table 2. When assessing the effects of ischaemia, only GLAST-sorted cells were considered. Flow cytometry data analysis was done with BD FACSDiva 8.0.2,

### Miniaturized scNMT-seq protocol

For profiling the transcriptome (cytoplasmic and nuclear mRNAs) and epigenome (DNA methylation and chromatin accessibility) of single cells, we developed and implemented a miniaturized and higher throughput version of the scNMT-seq protocol^13^. In this new version, the Smart-seq3^16^ method and specific normalization steps were implemented. A detailed version of the protocol is described in^17^. For some plates (see Supplementary Table 2) we used combinatorial indexing on the genomic DNA fraction, with a multiplexing capacity of 384 cells per run.

### Cre virus injection and ischaemia of *Dnmt3a*^*fl/fl*^ mice

To knock out *Dnmt3a* in cells of the striatum, we stereotactically injected 3 µl of AAV1_P5_Cre virus into both striata (coordinates calculated to bregma: anterior-posterior: 0 mm, medio-lateral: 2 mm, dorsal-ventral: 3 mm). Mice received 5.10 × 10^8^ viral genomes in 3 µl. BCCAO was performed in mice at 5 days post-injection and were sacrificed 2 dpi for quantification of PSA-NCAM^+^ cells. Injected naive mice as well as non-injected naive and post-ischaemic mice were used as control.

For statistical inference, we considered the proportion of PSA-NCAM^+^ neuroblasts among all live (Sytox Blue negative) cells. Inference was performed using a linear model fitted on the logit scale.

### Immunohistochemistry for YFP, DCX and TUNEL

Naive and 2 dpi ischaemic mice were used for immunohistochemistry to quantify YFP^+^ and DCX^+^ cells in the striatum (TiCY mice). Briefly, mice were perfused, and brains were fixed overnight with PFA 4%. Immunohistochemistry was performed on 50 µm vibratome sections as previously described^29^. The following antibodies were used: chicken anti-GFP (AVES, 1:500), guinea pig anti-DCX (Milipore, 1:100), goat anti chicken IgG Alexa 488 (ThermoFisher, 1:500), donkey anti guinea pig IgG Alexa 647 (Jackson Immunoresearch, 1:500). Cell death was analysed by TUNEL staining as described by the manufacturer (B6N mice, Click-iT TUNEL Alexa Fluor Imaging Assay). Nuclei (DAPI) and TUNEL^+^ nuclei were segmented with Cellpose 2.2.2^68^ and counted in the vSVZ and striatum. The proportion of TUNEL^+^ cells was averaged across technical replicates (slides) to obtain one value per biological replicate (individual mouse).

### Neurosphere assay

To assess the neurogenic potential of striatal and vSVZ cells upon ischaemia, a neurosphere assay was performed on the freshly isolated vSVZ and striatum of naive and post-ischaemic (2 dpi) B6N wild-type mice. Tissue dissociation was performed as previously described^29^. After dissociation and single-cell preparation, cells were plated in 96-well plates at a density of 1,000 cells in 200 µl per well. After 7 days, the number of wells that had neurospheres were counted. From the set of wells with neurospheres, a maximum of 10 wells were randomly chosen for quantifying the number and size of neurospheres. Three biological replicates were used for every condition.

### Processing of single-cell transcriptomic data

Transcriptomic reads were mapped to the mouse genome build GRCm38 (mm10) with STAR 2.7.3a^69^, using gene annotations downloaded from Ensembl^70^ Release 102. Both mapping and gene quantification were executed by the zUMIs pipeline 2.9.4f^71^ as described in the Smart-seq3 protocol (10.17504/protocols.io.bcq4ivyw).

### Processing of single-cell epigenomic data

Genomic reads were first trimmed with Trim Galore 0.4.4 (https://www.bioinformatics.babraham.ac.uk/projects/trim_galore/) in paired-end mode, and then mapped to GRCm38 with Bismark 0.22.3^72^ in single-end, non-directional mode. After filtering PCR duplicates with Bismark, single-end alignments were merged. DNA methylation at individual cytosines was quantified with Bismark’s coverage2cytosine script using the NOMe-seq option to distinguish between CpG and GpC contexts.

### Quality filtering and analysis of single-cell epigenomic data

CpG methylation and chromatin accessibility (GpC methylation) was analysed with MethSCAn 0.3.2, a command line tool enabling the analysis of single-cell methylation data that was developed in parallel to this study. For a detailed explanation of the statistical methods, see^21^. In brief, we used ‘methscan prepare’ to separately store CpG and GpC data in an efficient format and to compute quality metrics. Cells with read coverage of less than 50,000 CpG sites, or with poor methylation or accessibility profiles around their TSS were discarded. Methylation and accessibility profiles around TSSs and CTCF-binding sites were computed with ‘methscan profile’. When multiple TSSs were annotated for one gene we selected only the TSS of the ‘principal’ isoform, based on the APPRIS^73^ score in Ensembl release 102. CTCF-binding sites are based on CTCF ChIP-seq peaks downloaded from the ENCODE portal^74^ (accession number EENCFF242GNY). We used the ‘gimme scan’ command of GimmeMotifs 0.15.3^75^ to identify the exact position of CTCF motifs (from JASPAR2022^76^) within these peaks. We furthermore discarded low-quality cells according to a variable threshold on the number of observed genes (minimum 1,500). As reported in prior work using scNMT-seq^13,53^, some cells passed the RNA quality threshold but did not pass the methylome quality threshold. After filtering, we used ‘methscan smooth’ with a bandwidth of 1,000 (500 for GpC data) to quantify the smoothed mean methylation of all high-quality cells over the whole genome. VMRs and VARs were detected with ‘methscan scan’, a sliding window approach that scans the whole genome for regions of high methylation variance between cells. We used a bandwidth of 2,000 (1,000 for GpC data), a step size of 10 and a variance threshold of 0.2. We then quantified methylation and accessibility at VMRs, VARs and promoters (TSS ± 1,000 bp) using ‘methscan matrix’.

### Dimensionality reduction and pseudotime

We used Seurat 4.1.0^77^ to process the scRNA-seq data. To achieve higher resolution, we integrated our transcriptomic data with a much larger scRNA-seq dataset (wild-type cells from^18^) as previously suggested^53^. Specifically, after normalizing and finding 3,000 highly variable genes using default Seurat parameters for both datasets, we used FindIntegrationAnchors and IntegrateData using 30 dimensions to integrate the datasets, followed by scaling, principal component analysis (PCA) and UMAP on 30 principal components.

To visualize single-cell methylomes from naive mice, we subjected scaled and centred VMR methylation values to PCA, followed by UMAP on the top 15 principal components, excluding PC 5 which captured cell quality. Since epigenomic data contains missing values, we used a modified PCA that estimates missing values in an iterative manner^21^. To reduce noise, we used the ‘shrunken mean of residuals’ reported by ‘methscan matrix’ as a measure of methylation, and to reduce technical variation among cells we centred all values for each cell. Only VMRs observed in at least 20% of cells were used for PCA. This threshold did not strongly affect results (Supplementary Fig. 7). Accessibility data was processed in the same manner, with the following differences: Only VARs observed in at least 40% of cells were considered, and promoter accessibility values were used in addition to VAR accessibility.

To project information from all 3 molecular layers into a shared lower-dimensional space, we used MOFA+ 1.6.0^19^ aiming for 15 dimensions (factors). As input, we used the expression values of 3,000 highly variable genes (normalized with SCTransform 0.4.1^78^), as well as the same methylation and accessibility values previously used for PCA (promoter methylation and accessibility, VMR methylation, VAR accessibility). We used UMAP on the top 13 MOFA factors, excluding factors 4 and 11, which captured technical variation. We then used Leiden clustering^79^ on the MOFA factors, followed by slingshot 2.4.0^80^ to obtain pseudotime values informed by both gene expression and epigenetics. Oligodendrocytes were excluded from pseudotime analysis since the focus of our study is on neurogenesis.

The above section describes the analysis of samples from naive mice. To compare naive and post-ischaemic samples, we repeated the dataset integration using all available single-cell transcriptomes from this study and ref. ^18^ as described above. The same procedure was repeated to integrate additional naive striatal and cortical astrocytes which were sequenced at a later date. Leiden clusters and pseudotime (using slingshot) were re-calculated on the integrated transcriptome PCA.

### Correlation of epigenetic features with gene expression

Promoter methylation (mean shrunken residuals) and log-normalized gene expression values were correlated and tested for significance with the R function cor.test (two-sided), using Pearson correlation. VMR methylation was correlated with the expression of the closest gene, as determined with bedtools 2.30.0^81^: bedtools closest -D ‘b’ -a regions.bed -b gene_bodies.bed. Accessibility was correlated in the same manner. Only regions with genomic reads in at least five cells were considered. Correlation *P* values were adjusted for multiple testing with the Benjamini–Hochberg method.

We used ‘methscan matrix’ to quantify DNA methylation (shrunken mean of residuals) in 3,000-bp-wide intervals downstream of TSSs. Specifically, we considered the TSSs of all protein-coding genes and used the interval from +2 kb downstream to +5 kb downstream of each TSS. TSSs with sequencing coverage in at least 5 cells per group were then tested for differential methylation between astrocytes (vSVZ, striatum) and NSC-lineage cells (qNSC2→neuroblast) with the two-sided Wilcoxon rank sum test. The same approach was applied to log-normalized RNA counts to determine genes up- or downregulated in the NSC lineage. Both sets of Wilcoxon *P* values were adjusted for multiple testing with the Benjamini–Hochberg method. TSSs were then binned according to the rounded methylation difference, counting TSSs with an adjusted Wilcoxon *P* value > 0.05 or a methylation difference smaller than 5% as not significant.

ChIPseeker 1.32.0^82^ was used to quantify the number of VMRs and VARs that overlap with gene features, using the options “tssRegion=c(−1000, 1000)” and “overlap=‘all’”. Overlaps with candidate *cis*-regulatory elements (cCREs, Registry V3, downloaded from https://screen.encodeproject.org/ on 3rd August 2021^83^) were quantified with the mergeByOverlaps function of the GenomicRanges (1.48.0) R package^84^.

### Quantifying methylation change along pseudotime

Correlation heat maps of each molecular layer were generated either by grouping cells by cell state, or by binning cells along pseudotime with a mean of ten cells per bin. For all binned heat maps of non-ischaemic cells, we enforced that each bin only contains cells from one cluster and tissue, so that—for example, the first cluster contains only striatal astrocytes. Methylation, accessibility, and expression values were averaged per cell state or bin and the Pearson correlation of all bins was visualized with ComplexHeatmap 2.12.0^85^. We used the ward.D2 method of the R function hclust for hierarchical clustering of cell states. We chose to omit the cell state correlation heat map for chromatin accessibility data since the results depended greatly on the choice of pre-processing methods.

To quantify (de)methylation events in the NSC lineage, we considered all VMRs that were observed in at least 100 cells of the naive wild-type NSC lineage including vSVZ astrocytes. For each VMR, we fit a step function to the methylation values as a function of pseudotime. The function is parametrized by a change point *s* in pseudotime and two constant values, which the function takes before and after *s*. Minimizing the sum of squared residuals over this parameter space, we found a most likely value for the methylation change point in pseudotime. VMR change points were considered (de)methylation events if the step function fit was at least 15% better (with respect to the squared residuals sum) than a constant fit without a step. To visualize expression, methylation and chromatin accessibility of genes affected by demethylation in late TAPs (the ‘second wave’), we selected VMRs with an inferred change point between pseudotime ranks 250 and 400 that intersect with a gene. For each of these VMRs, we visualized VMR methylation, log-normalized expression of its intersecting gene, and VMR accessibility in heat maps.

### Epigenetic changes near cell type-specific genes

Representative marker genes for each cell type or stage were determined with the two-sided Wilcoxon rank sum test, by testing log-normalized expression values in cells of interest against the expression values of all other cells. We selected the top 100 most differentially expressed genes among genes with a Benjamini–Hochberg-adjusted *P* value below 0.05 that also contain a VMR in their gene body. Expression, methylation and accessibility values of these genes and their corresponding promoters or VMRs were averaged.

### Transcription factor motif enrichment

Since the PCA on VMR methylation values captured methylation differences between oligodendrocytes and the neurogenic lineage on the first principal component (PC1), we selected the 5,000 VMRs with the highest PC1 loading for oligodendrocyte-specific motif enrichment. We used HOMER 4.4^61^ with the Jaspar2022 motif database^76^ to identify motifs enriched in these VMRs:


findMotifsGenome.pl VMRs.bed mm10r output/ -len 5,6,7,8,9,10,11,12 -size given -mcheck JASPAR.db -mknown JASPAR.db


The same strategy was used to identify motifs enriched in regions with low methylation in the neurogenic lineage (5,000 VMRs with the highest PC2 loading) and in common parenchymal astrocytes (lowest 5,000 PC2 loadings).

### Identification of LMRs and associated GO terms

To identify regions that are differentially methylated between two groups of non-ischaemic cells, we compared VMRs with the two-sided Wilcoxon rank sum test. Only VMRs with genomic coverage in at least 30 cells per group were considered. VMRs with a Benjamini–Hochberg-adjusted *P* value below 0.05 were labelled LMRs. For visualizing gene expression in volcano plots and heat maps, all LMRs overlapping a gene body were assigned to that gene. We used GREAT 4.0.4^62^ for GO term enrichment of genes near LMRs, using the option “basal plus extension” with a constitutive 20 kb downstream regulatory domain and up to 1,000 kb maximum extension.

To visualize smooth methylation tracks of LMRs and their surroundings, we averaged CpG methylation values in pseudobulk cell groups and smoothed these means with a weighted kernel smoother (tricube kernel, 1,000 bp bandwidth).

### Expression and methylation signatures induced by ischaemia

Reactive astrocyte marker gene sets^46,47^ were used to calculate expression signatures (mean log-normalized expression of the respective gene set). We used the ‘Pan reactive’, ‘A1 specific’ and ‘A2 specific’ gene sets from ref. ^46^. We used the lipopolysaccharide-induced, cluster-specific differential expression results reported in ref. ^47^ to determine differentially expressed genes that are either shared among all astrocyte clusters (consistently lipopolysaccharide-induced), or only in one specific cluster (as in fig. 3c in ref. ^47^). NSC methylomes and astrocyte methylomes were distinguished based on the mean methylation of all astrocyte and NSC LMRs; the depicted methylation score is the difference of these two means.

To detect DMRs induced by ischaemia, we selected all qNSC2 cells, aNSCs and TAPs from the vSVZ. We then used the command ‘methscan diff --bandwidth 2000 --stepsize 100 --threshold 0.05 --min-cells 6’ to test cells from 2 dpi against cells from naive mice^21^. This approach was repeated to test 21 dpi cells against naive. Data exploration and visualization was done in R/tidyverse 1.3.1.

### Reporting summary

Further information on research design is available in the Nature Portfolio Reporting Summary linked to this article.

## Online content

Any methods, additional references, Nature Portfolio reporting summaries, source data, extended data, supplementary information, acknowledgements, peer review information; details of author contributions and competing interests; and statements of data and code availability are available at 10.1038/s41586-024-07898-9.

## Supplementary information

Supplementary Information This file contains Supplementary Figs. 1–7.

Reporting Summary

Supplementary Table 1 Cell metadata. Quality metrics and other metadata of 2584 cells, isolated from a total of 15 biological replicates.

Supplementary Table 2 Sequencing experiments. Detailed information on all sequencing experiments, including experimental dates, mouse genotypes, treatments and the total number of biological replicates.

Supplementary Table 3 Marker genes. List of marker genes used for the heat maps shown in Extended Data Fig. 2d.

Supplementary Table 4 LMRs. List of astrocyte LMRs and NSC LMRs and their nearest genes.

## Source data

Source Data Fig. 5

Source Data Extended Data Fig. 8

## Data Availability

All sequencing data are available under GEO accessions GSE209656 (single-cell transcriptomes) and GSE211786 (single-cell epigenomes). The external scRNA-seq dataset used for transcriptome integration is available under GEO accession GSE197217. CTCF-binding sites are from ENCODE experiment ENCFF242GNY. The following databases/datasets were used: mouse genome GRCm38, Ensembl release 102 mouse genome annotation, ENCODE cCRE Registry V3, JASPAR TFBS database release 2022. Source data are provided with this paper.

## Code availability

The software that we developed to analyse single-cell methylome data is free and open source, and available at https://anders-biostat.github.io/MethSCAn. Additional scripts specific to this study are deposited at https://github.com/LKremer/scbs/tree/astrocyte-stemness-analysis.
