# Supplementary material for: DNA methylation controls stemness of astrocytes in health and ischaemia
Source: Nature. 2024 Sep 4;634(8033):415–23. doi: 10.1038/s41586-024-07898-9 (PMC11464379; doi:10.1038/s41586-024-07898-9)
Supplement: Supplementary file 1 — This file contains Supplementary Figs. 1–7. [file 41586_2024_7898_MOESM1_ESM.pdf]

nature portfolio

https://doi.org/10.1038/s41586-024-07898-9

Supplementary information

DNA methylation controls stemness of astrocytes in health and ischaemia

In the format provided by the authors and unedited

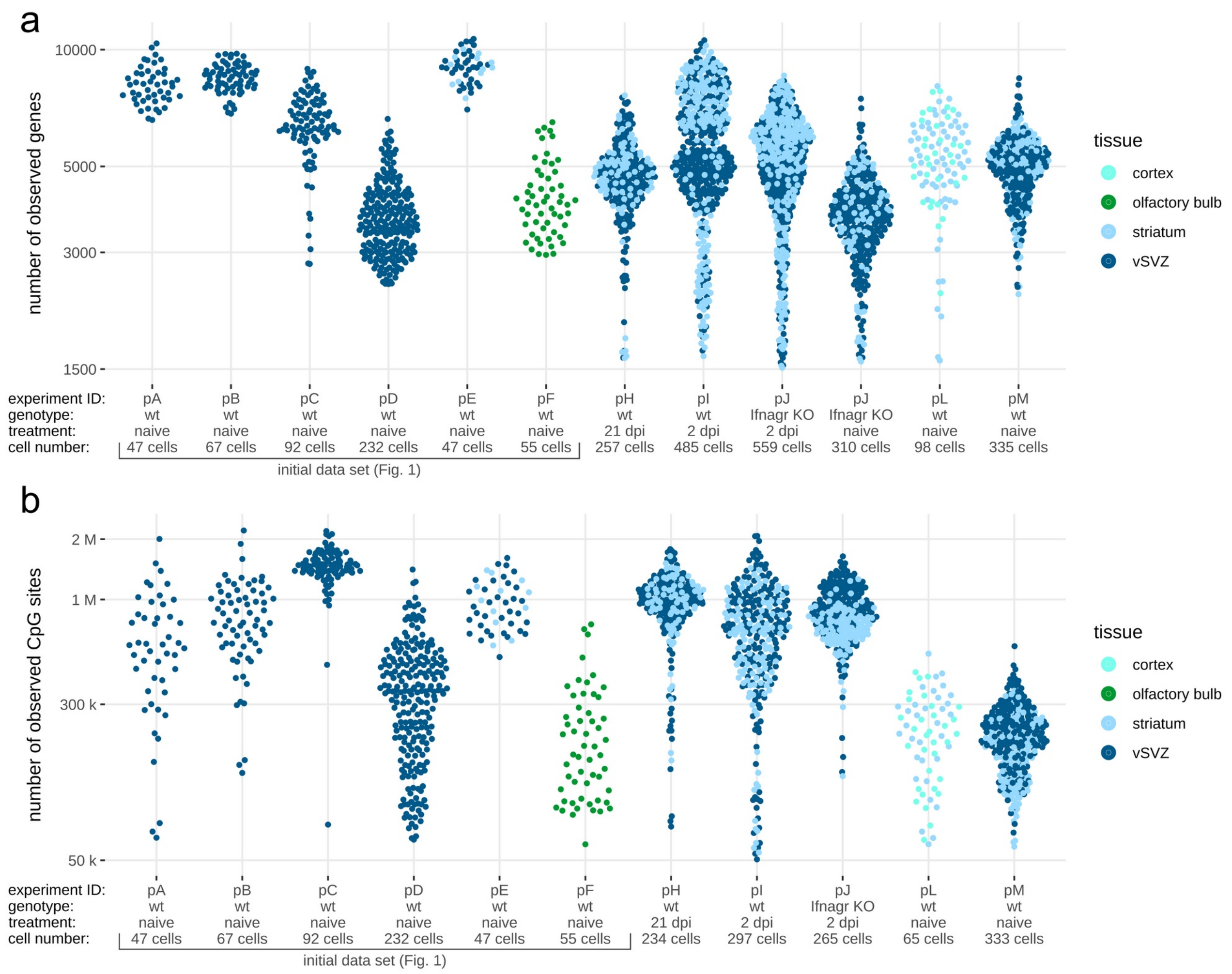

Supplementary Figure 1: Quality metrics of scNMT-seq cells that passed methylome quality filtering.

a, Number of observed genes per cell, shown for each experiment. Depicted are n=1734 cells from 15 biological replicates (some experiments comprise multiple mice separated on the plate, see Supplementary Tables 1-2 for details). b, Number of observed CpG sites per cell. Quality threshold: 50.000 CpG sites. For the initial data set shown in Fig. 1, we only considered cells that passed both quality thresholds. For later experiments (pH - pM), we also considered cells that only passed the gene threshold for transcriptome analysis only. Only cells isolated based on their expression of the GLAST surface marker (all except pA, pB, pC and pF) were included in the comparisons between naive and ischemia.

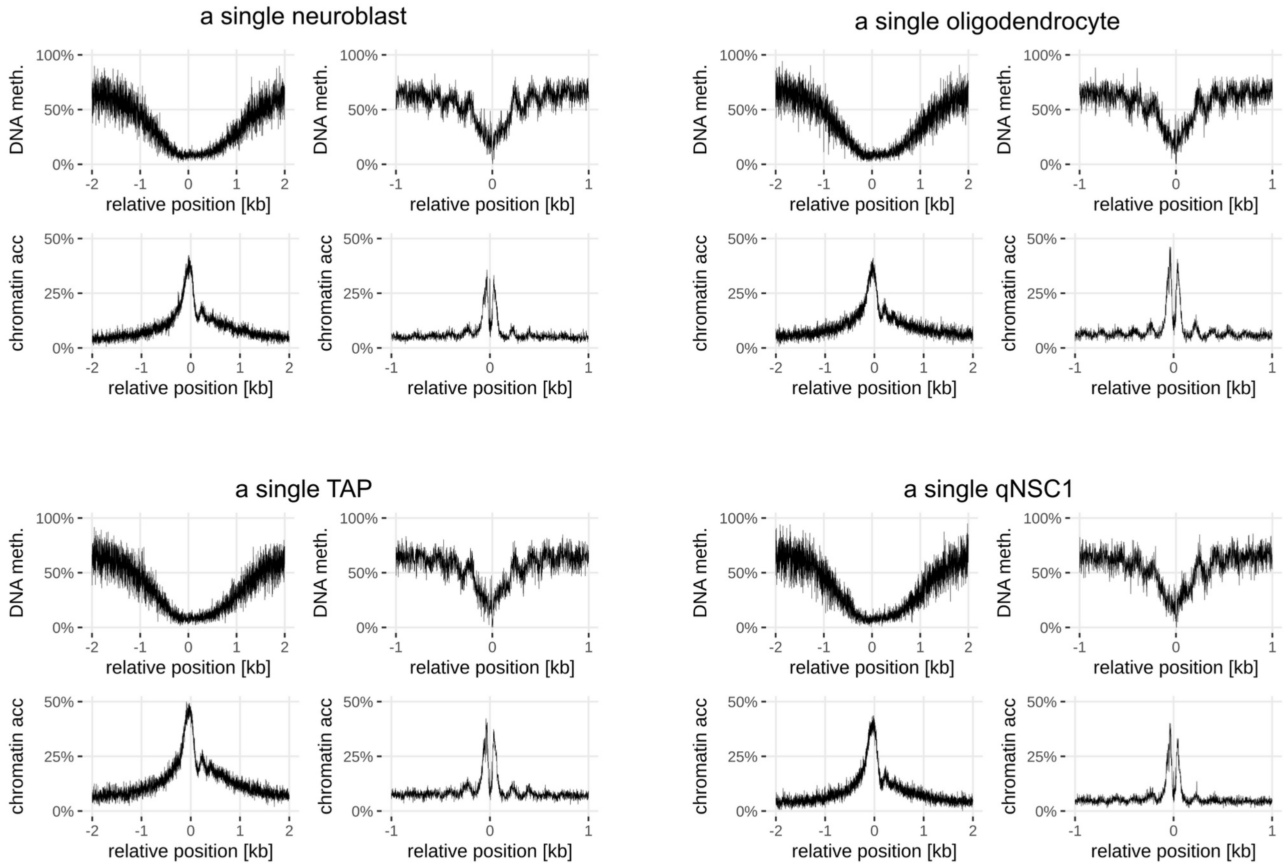

Supplementary Figure 2: Average methylation and chromatin accessibility levels around transcription start sites (TSS) and CTCF-binding sites of four individual cells.

For each of the four depicted cells, the average TSS profile is shown on the left (top: CpG methylation, bottom: accessibility measured via GpC accessibility) and the average CTCF profile is shown on the right. These plots are based on the initial naive wild type data set highlighted in Supplementary Fig. 1, which comprises n=540 cells from 8 biological replicates (see Supplementary Tables 1-2 for details).

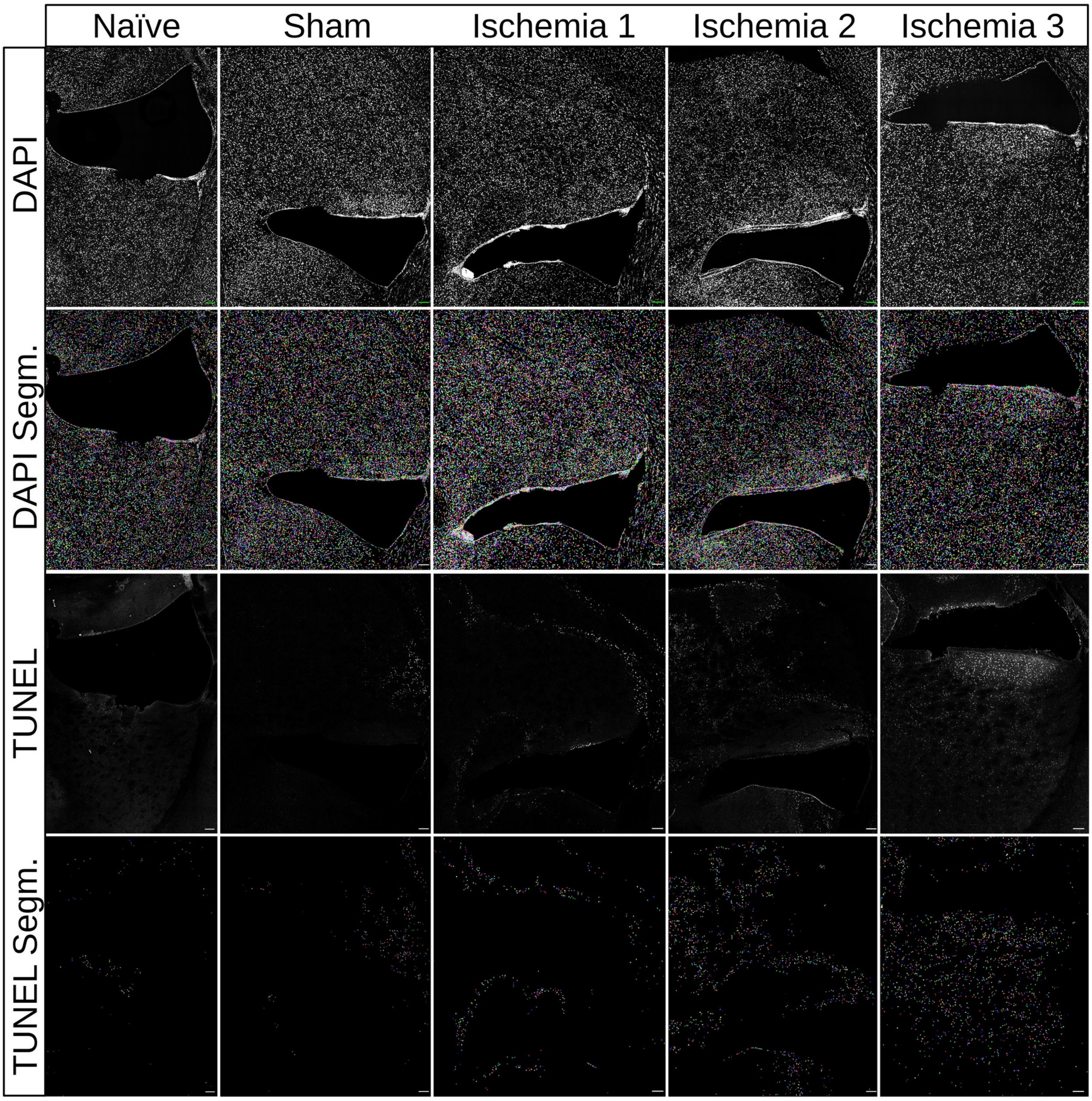

Supplementary Figure 3: TUNEL staining to detect apoptosis 2 days post ischemia.

Confocal images of subventricular zones and striatum of three mice isolated two days post ischemia, one sham operated mouse and one naive control mouse. 1st row: DAPI; 2nd row: DAPI segmented; 3rd row: TUNEL; 4th row: TUNEL segmented. Scale bar 100 µm.

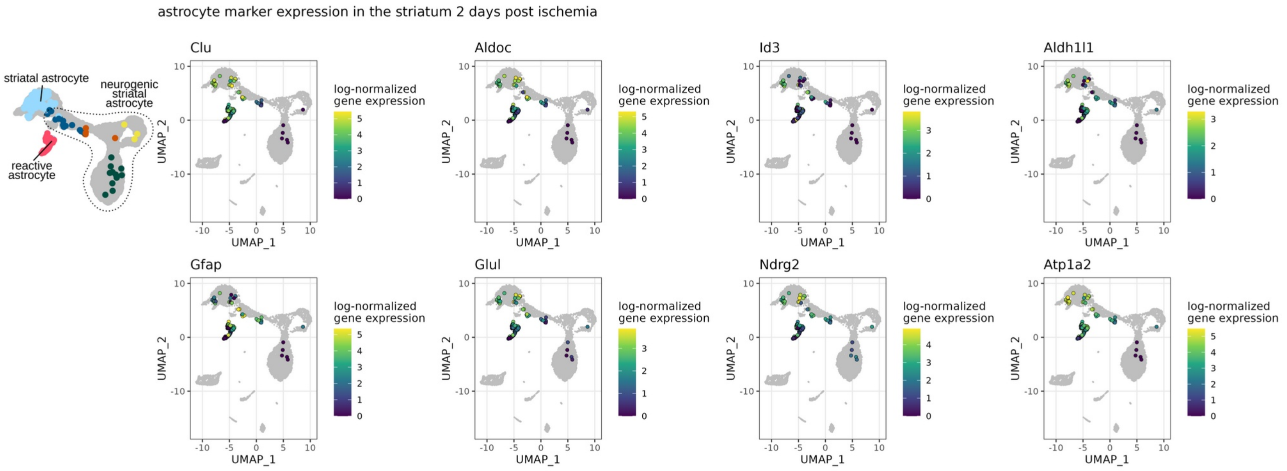

Supplementary Figure 4: Expression of astrocyte marker genes in the striatum two days post ischemia.

The UMAP location of cell states is depicted in the top left. Note that most markers are still expressed in striatal astrocytes that have entered the neurogenic lineage (highlighted by the black outline in the top left legend).

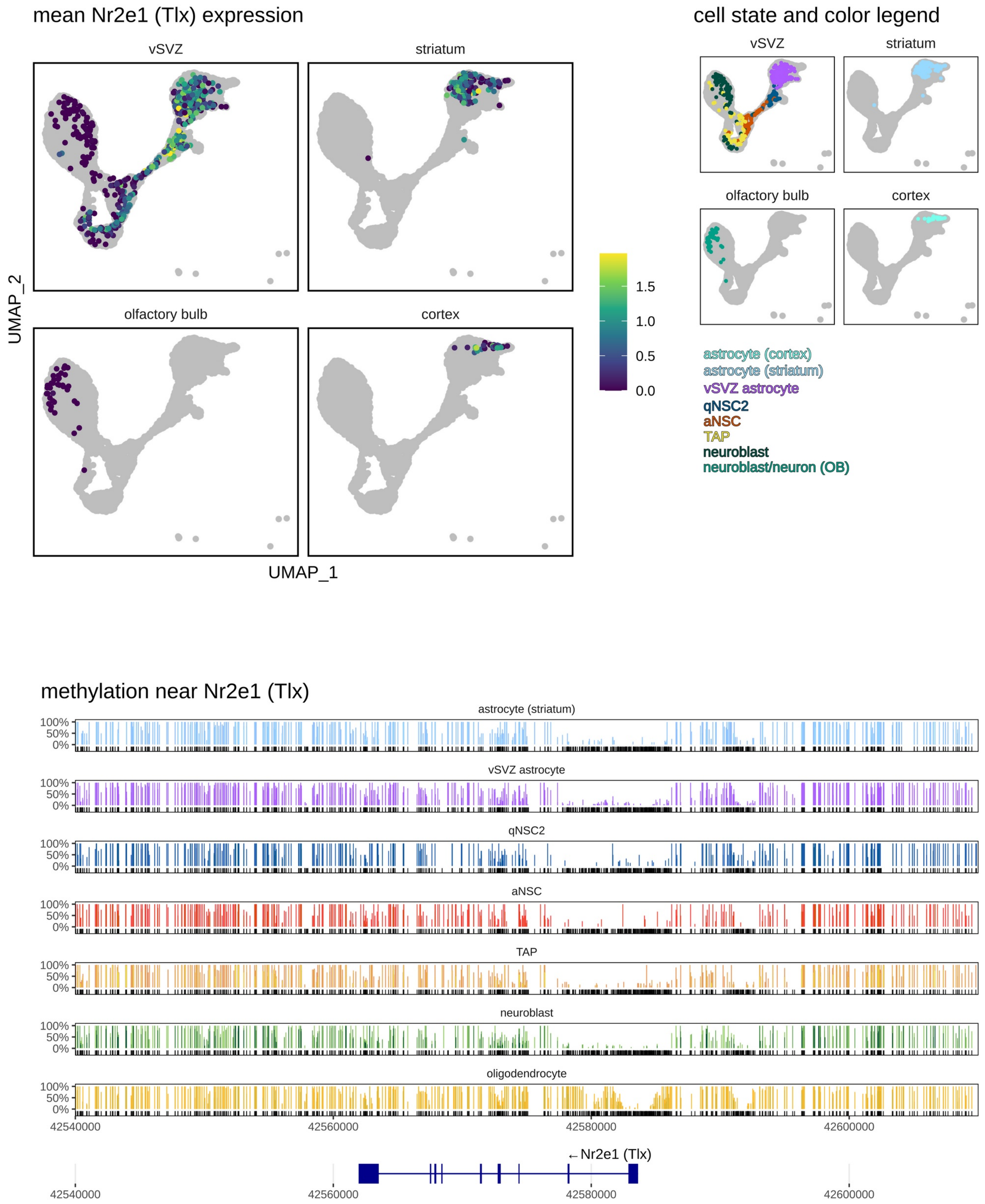

Supplementary Figure 5: Expression and DNA methylation of the NSC marker *Nr2e1* (*Tlx*) in cells isolated from naive brains.

Top left: transcriptome UMAPs, cells are colored by log-normalized Nr2e1 expression. Bottom: pseudobulk DNA methylation tracks near *Nr2e1*. As previously reported (Magnusson et al., 2020, https://doi.org/10.7554/eLife.59733) this marker also shows some mRNA expression in astrocytes outside the vSVZ.

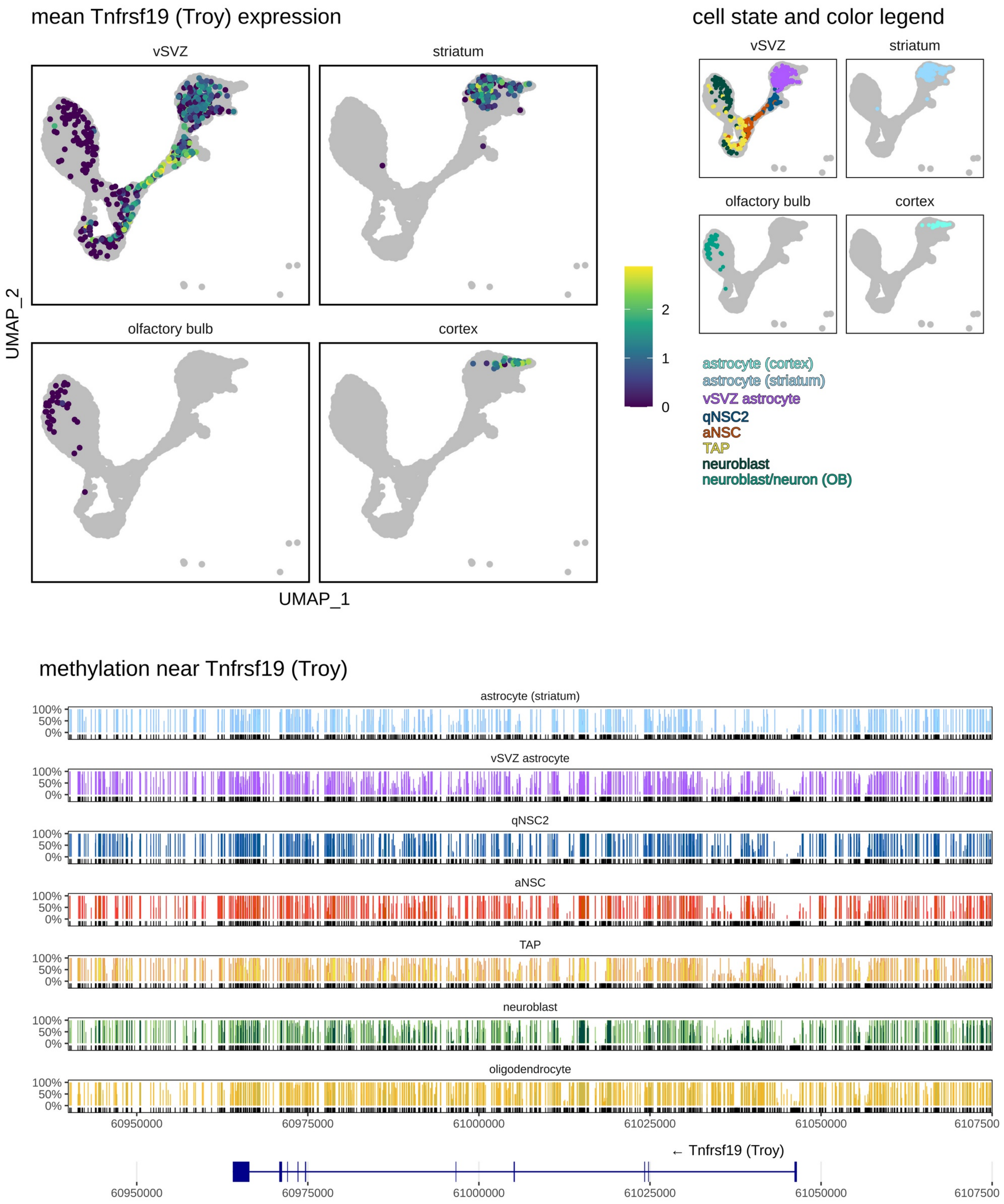

Supplementary Figure 6: Expression and DNA methylation of the NSC marker *Tnfrsf19* (*Troy*) in cells isolated from naive brains.

Top left: transcriptome UMAPs, cells are colored by log-normalized Tnfrsf19 expression. Bottom: pseudobulk DNA methylation tracks near *Tnfrsf19*. As previously reported (Magnusson et al., 2020, https://doi.org/10.7554/eLife.59733) this marker also shows some mRNA expression in astrocytes outside the vSVZ.

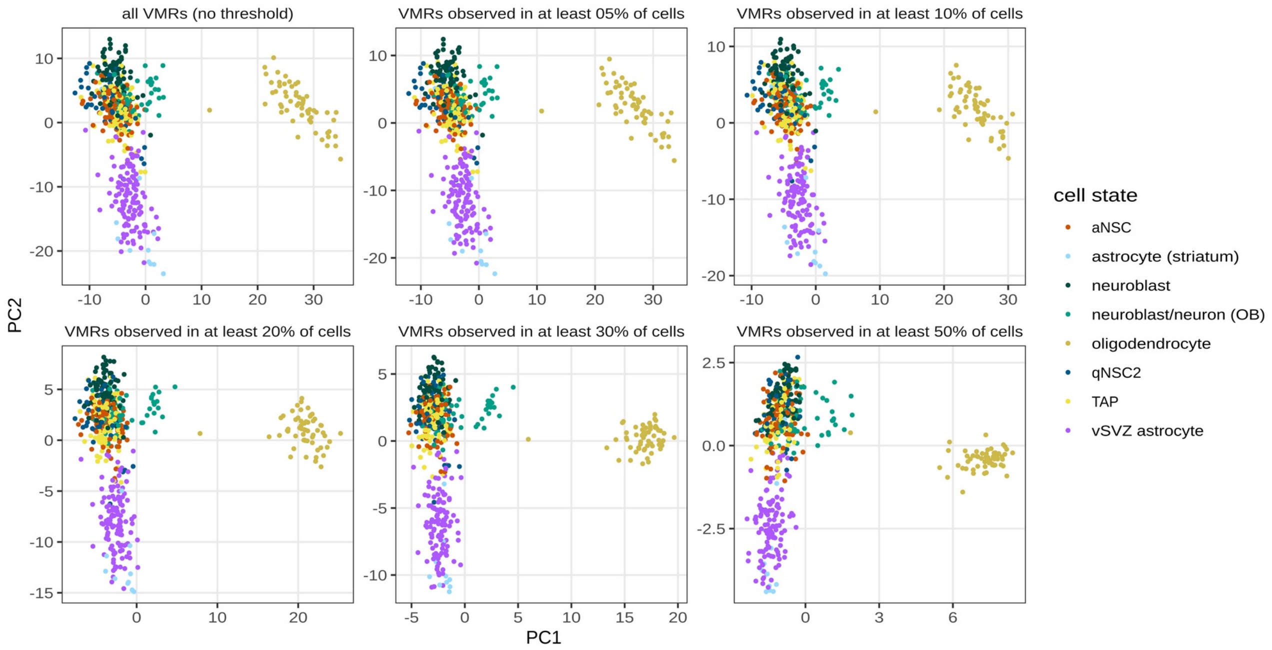

Supplementary Figure 7: PCA of single cell methylation data after applying different feature quality-filtering thresholds.

The depicted cells are from naive wild type mice. Top left: PCA based on CpG methylation (shrunken mean of residuals from the MethSCAn tool) of all detected VMRs (variably methylated regions). These VMRs were subjected to quality filtering by discarding those VMRs that did not have sequencing coverage in a certain proportion of cells. The ability to separate cell types in PC space is not strongly affected by the exact choice of quality filtering threshold.
